# MacroH2A1 associates with nuclear lamina and maintains chromatin architecture in mouse liver cells

**DOI:** 10.1038/srep17186

**Published:** 2015-11-25

**Authors:** Yuhua Fu, Pin Lv, Guoquan Yan, Hui Fan, Lu Cheng, Feng Zhang, Yongjun Dang, Hao Wu, Bo Wen

**Affiliations:** 1 Key Laboratory of Molecular Medicine of Ministry of Education and Institutes of Biomedical Sciences, Shanghai Medical College, Fudan University, Shanghai 200032, China; 2 Department of Chemistry, Fudan University, Shanghai, 200433; 3 State Key Laboratory of Genetic Engineering and Collaborative Innovation Center of Genetics and Development, School of Life Sciences, Fudan University, Shanghai 200438, China; 4 Department of Biostatistics and Bioinformatics, Emory University, Atlanta, GA 30322, USA

## Abstract

In the interphase nucleus, chromatin is organized into three-dimensional conformation to coordinate genome functions. The lamina-chromatin association is important to facilitate higher-order chromatin in mammalian cells, but its biological significances and molecular mechanisms remain poorly understood. One obstacle is that the list of lamina-associated proteins remains limited, presumably due to the inherent insolubility of lamina proteins. In this report, we identified 182 proteins associated with lamin B1 (a constitutive component of lamina) in mouse hepatocytes, by adopting virus-based proximity-dependent biotin identification. These proteins are functionally related to biological processes such as chromatin organization. As an example, we validated the association between lamin B1 and core histone macroH2A1, a histone associated with repressive chromatin. Furthermore, we mapped Lamina-associated domains (LADs) in mouse liver cells and found that boundaries of LADs are enriched for macroH2A. More interestingly, knocking-down of macroH2A1 resulted in the release of heterochromatin foci marked by histone lysine 9 trimethylation (H3K9me3) and the decondensation of global chromatin structure. However, down-regulation of lamin B1 led to redistribution of macroH2A1. Taken together, our data indicated that macroH2A1 is associated with lamina and is required to maintain chromatin architecture in mouse liver cells.

The nuclear lamina (NL) is a filamentous meshwork underneath the inner nuclear membrane. Besides its structural role to support nucleus, NL is involved in various cellular processes such as DNA replication^1^, transcription repression^2^, RNA processing^3^,^4^,^5^ and chromatin organization^6^. Furthermore, genetic alterations of lamina components have been linked to more than 20 diseases, but the disease mechanisms remain largely unknown^7^.

In the interphase nucleus, the positioning of chromatin and regulatory factors is not random, but associated with specific nuclear compartments such as nuclear periphery, thereby coordinating genome functions^8^,^9^. For example, about 40% of mammalian genome is organized into lamina-associated domains (LADs)^6^, significantly overlapping the large repressive chromatin domains enriched for H3 lysine 9 dimethylation (H3K9me2)^10^,^11^. Recent studies indicated that several proteins, such as G9a, HDAC3, YY1 and Lamin A/C, are required to regulate the association between chromatin and nuclear lamina^9^,^12^. However, the biological functions of NL-chromatin association in development and diseases remain obscure, and their molecular mechanisms are still poorly understood.

The primary components of nuclear lamina are lamins, a family of type V intermediate filament proteins. There are two major types of lamin proteins: A-type (lamin A/C) and B-type (lamin B1 and B2). In mammals, lamin A and C are encoded by one gene *LMNA*, whereas two separate genes, *LMNB1* and *LMNB2*, encode lamin B1 and B2. Lamin A/C is developmentally regulated, and is absent or slightly expressed in some cell types such as embryonic stem cells^13^. By contrast, almost all type of cells express B-type lamins. Depletion of lamin B1 and B2 resulted in abnormal organogenesis in the mouse, although the self-renewal of embryonic stem cell was not affected^14^. Interestingly, A- and B-type lamins form distinct nuclear compartments in mammalian cells and have specific roles for chromatin organization and gene expression^15^. These data suggest that different types of cells utilize different combinations of lamins and their associated proteins to execute cellular functions of lamina^16^.

Although the importance of lamins has been appreciated for decades, their protein networks are still largely unknown. Unlike nucleoplasmic proteins, lamins are strongly resistant to detergents and salts, and this inherent insolubility make it is extremely difficult to be analyzed by conventional biochemical approaches^17^. The recently developed proximity-dependent biotin identification (BioID) shows considerable promise for overcoming the issue of insolubility^18^. By using BioID, Roux *et al.* have identified 122 protein candidates associated with lamin A in Hela cells^18^. However, proteomes associated with lamin B have not been investigated systematically.

In this report, we developed vectors for lentivirus-based BioID assay, and generated the first proteome associated with lamin B1 (LMNB1) in mouse hepatocytes. As the first step to characterize these proteins, we found that histone variant macroH2A1 is associated with lamina and is essential to maintain chromatin architecture in mouse liver cells.

## Results

### LMNB1-associated proteins in mouse hepatocytes

**Figure 1:**
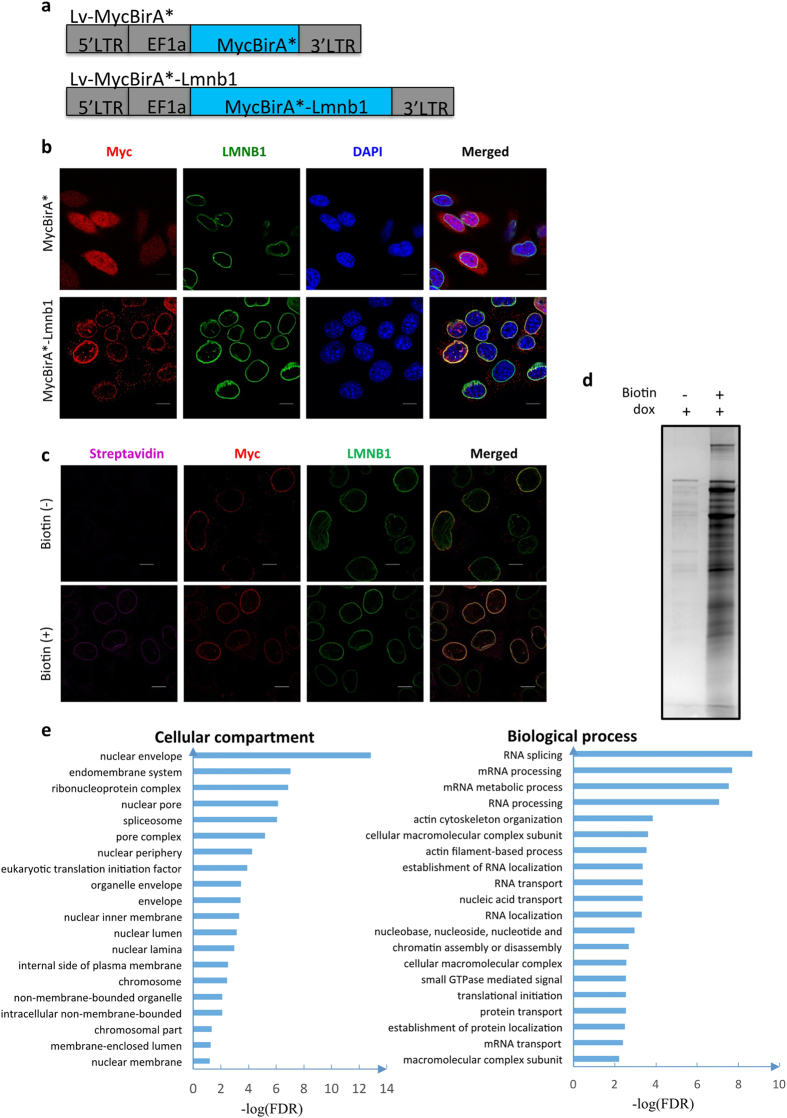
Identification of proteins associated with LMNB1 in AML12 cells by virus-based BioID assay. (**a**) Schematic drawing of vectors with the lentiviral backbone: Lv-MycBirA* and Lv-MycBirA*-Lmnb1. LTR = long terminal repeat. EF1a is the promoter of fusion proteins. (**b**) Localization of fusion proteins in AML12 cell infected with lentivirus particles. In the immunofluorescence experiments, exogenous fusion proteins and LMNB1 were detected with anti-myc (red) and anti-LMNB1 (green) antibodies, and DNA was labeled with DAPI (blue). Myc-BirA* was used as random control. Scale = 10 μm. (**c**) Localization of biotinylated proteins in AML12 cells infected with lentivirus. Biotinylated proteins were detected with streptavidin (purple); exogenous fusion proteins were detected with anti-Myc (red) antibody. Scale = 10 μm. Stained cells were imaged with the confocal laser scanning microscopy. (**d**) Biotinylated proteins as visualized by silver-stained SDS-PAGE. BioID experiments were conducted in AML12 cells infected with lentivirus of Lv-MycBirA*-Lmnb1. For the samples with (+) or without (−) dox, equal amount of cells were used for BioID experiments, and same volumes of eluted products were loaded on the gel. (**e**) Top twenty Gene Ontology (GO) terms of cellular compartments and biological process ranked by false discover rates (FDR). The list of LMNB1-associated proteins (Supplementary Table S1) was used for GO analysis with the DAVID tools.

To analyze LMNB1 associated proteins in non-cancer cell lines which preserve more intact nuclear architecture, we engineered BirA* element into an induced lentiviral vector and constructed the Lv-MycBirA* and Lv-MycBirA*-Lmnb1 plasmids (Fig. 1a). To test these plasmids, we transduced virus particles in AML12 cells, a mouse hepatocyte cell line with well-patterned nuclear architecture^19^. The results of Immunofluorescence (IF) with Myc and LMNB1 antibodies indicated that the MycBirA*-LMNB1 fusion protein locates on nuclear periphery and largely co-localizes with endogenous LMNB1 proteins, whereas the localization of MycBirA* was ubiquitous (Fig. 1b. After the cells were exposed to 50μM of biotin for 8 hours, biotinylated proteins were detected with fluorescent streptavidin, and the results showed that most of biotinylated proteins were associated with LMNB1 (Fig. 1c). These data indicated that the BirA*-LMNB1 fusion proteins are functional and distributed on the nuclear periphery.

We then carried out virus-based BioID experiments in AML12 cells. Biotinylated proteins were captured with streptavidin beads and visualized by silver staining. Compared to the Biotin minus control, many protein bands were appeared in the presence of Biotin (Fig. 1d). The captured products were analyzed by mass spectrometry in three independent experiments. In total, 182 proteins were identified with high confidence (Supplementary Table S1).

To seek biological insight into the LMNB1-associated proteins, we conducted Gene Ontology (GO) analysis. These proteins are enriched on cellular compartments such as nuclear envelope, spliceosome and chromosome, and are associated with biological processes including RNA splicing and chromatin assembly (Fig. 1e).

### Core histone variant macroH2A1 associates with LMNB1

**Figure 2:**
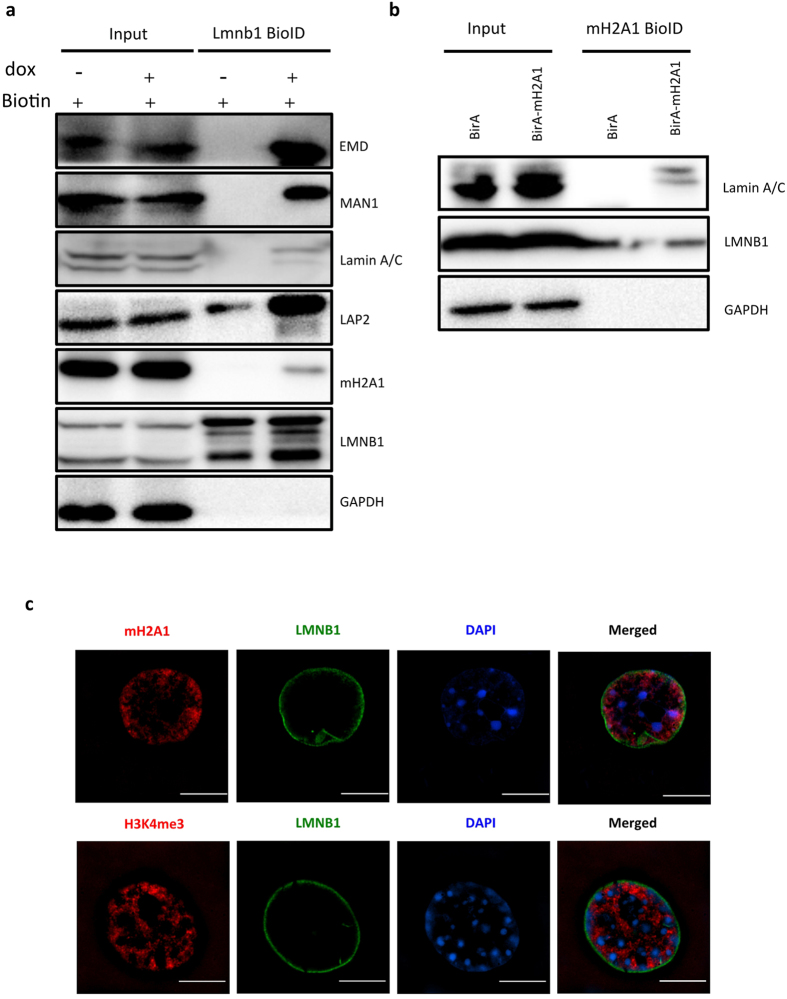
LMNB1 associates with histone variants macroH2A1. (**a**) Validation of proteomic data with western blotting. Whole cell extract (Input) and captured biotinylated proteins (BioID) were blotted with the indicated antibodies, in presence of biotin. Starting materials were equal for the samples with (+) and without (−) dox. GAPDH was used as a negative control. (**b**) Test of macroH2A1-LMNB1 association by BioID using macroH2A1 as the bait. Whole cell extract (Input) and macroH2A1 BioID products in Hela cells were blotted with the indicated antibodies. GAPDH was used as a negative control. (**c**) Sub-cellular localization of macroH2A1 or H3K4me3 (red) and LMNB1 (green) were shown by immunofluorescence and super-resolution microscope (SIM) in AML12. DNA was labeled with DAPI (blue). Scale = 10 μm.

To validate the proteomics data, we examined the BioID products by western blotting, and confirmed the LMNB1-association of four proteins in nuclear envelope (MAN1/LEMD3, Lamin-A/C, LAP2/TMPO, EMD) and macroH2A1 (mH2A1), a histone variant associated with repressive chromatin (Fig. 2a). Furthermore, we constructed BirA*-*macroH2A1* plasmid, and transiently transfected it in Hela cells. We then performed BioID experiment and tested the captured biotinylated proteins by western blot. As expected, both lamin A/C and LMNB1 can be detected by western blot, whereas there was no signal for GAPDH (Fig. 2b). These data indicate that macroH2A1 and LMNB1 are physically associated.

Since the proteins detected by BioID could be interacting with the bait directly or indirectly, we further deciphered subcellular localization of macroH2A1 by super-resolution Structured Illumination Microscopy (SIM) in AML12 cells. Surprisingly, macroH2A1 signals are ring-like and distributed near nuclear periphery, whereas the signals of H3K4me3, an active histone modification, mainly locate in the nuclear interior (Fig. 2c). Notably, the signals of macroH2A1 (red) and LMNB1 (green) are not overlapped (Fig. 2c), suggesting that their association could be indirect.

### MacroH2A is enriched on boundaries of Lamina-associated domains

To analyze lamina-chromatin associations in liver cells, we performed DNA adenine methyltransferase identification (DamID) experiments and the LMNB1 associated DNA fragments were detected with microarrays. Four biological replicates were conducted and the average correlation among samples is 0.85, indicating a good reproducibility of these experiments (Figure S1). Based on the four replicates, we detected LADs using probe level t-statistics (Figure S1 and Method), and identified 1,317 in AML12 cells (Supplementary Table S2). These LADs cover 48.3% of the mouse genome, and the median length of LADs is 597 kb. The global pattern of NL-chromatin interactions in liver cells is generally similar to those in other cell types (Figure S3).

**Figure 3:**
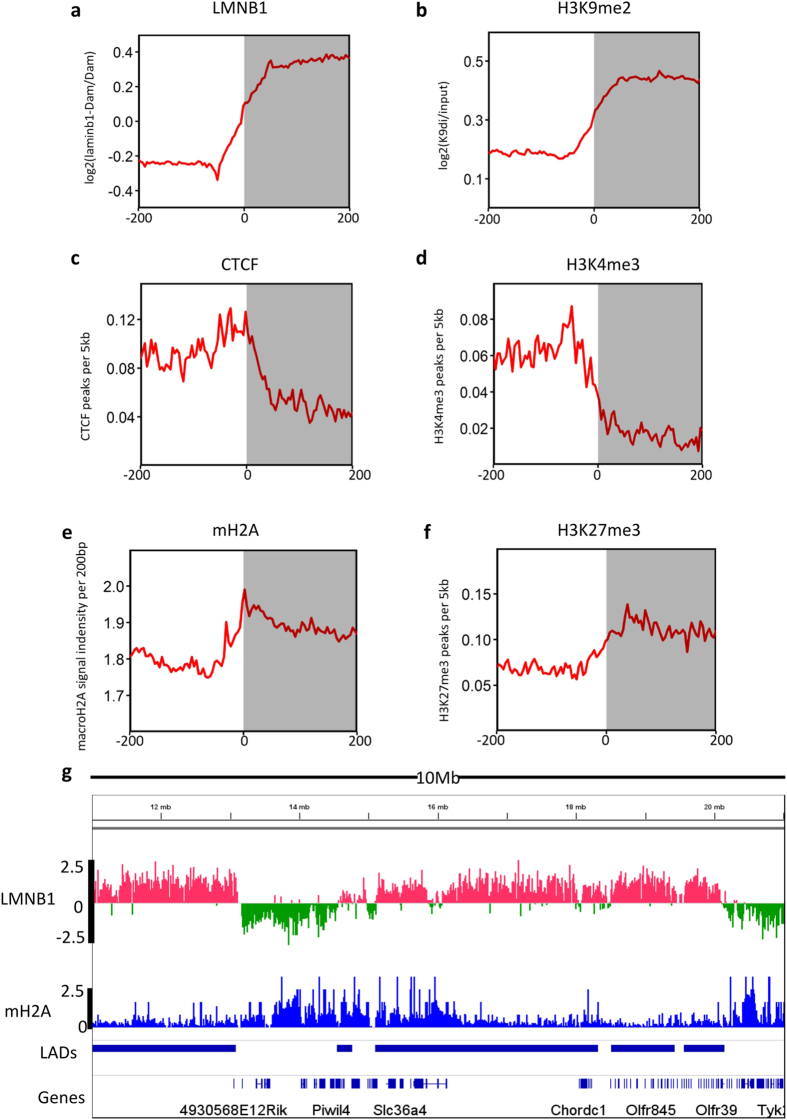
MacroH2A is enriched on boundaries of Lamina-associated domains. (**a**–**f**) Profiles of chromatin features aligned against LAD border regions (200 kb inside and outside LADs). The X-axis represents relative positions to the nearest boundary, and the Y-axis represents averaged binding ratios (LMNB1, H3K9me2), averaged number of peaks (H3K4me3, H3K27me3, CTCF), and averaged signal intensity (macroH2A). Gray areas indicate regions inside LADs, and red lines show averages with moving window sizes of 5 kb. (**b**) As an example, a 10 Mb region along chromosome 18 showing the enrichment of macroH2A around the LAD borders.

We then analyzed chromatin patterns on boundaries of LADs in mouse liver cells. Like lamin B1 (Fig. 3a), repressive histone modifications H3K9me2 are highly enriched within the LADs (Fig. 3b). By contrast, the active promoter mark H3K4me3, and the insulator CCCTC-binding factor (CTCF) are enriched at the boundaries outside the LADs (Fig. 3c,d). Surprisingly, the average macroH2A occupancy is more presented inside LADs, and forms a peak on the boundary (Fig. 3e), similar to that of H3K27me3 (Fig. 3f). For example, on a 10 Mb region of chromosome 18 (Fig. 3g), macroH2A is enriched on the boundaries of LADs.

### Down-regulation of macroH2A1 reshapes chromatin architecture

**Figure 4:**
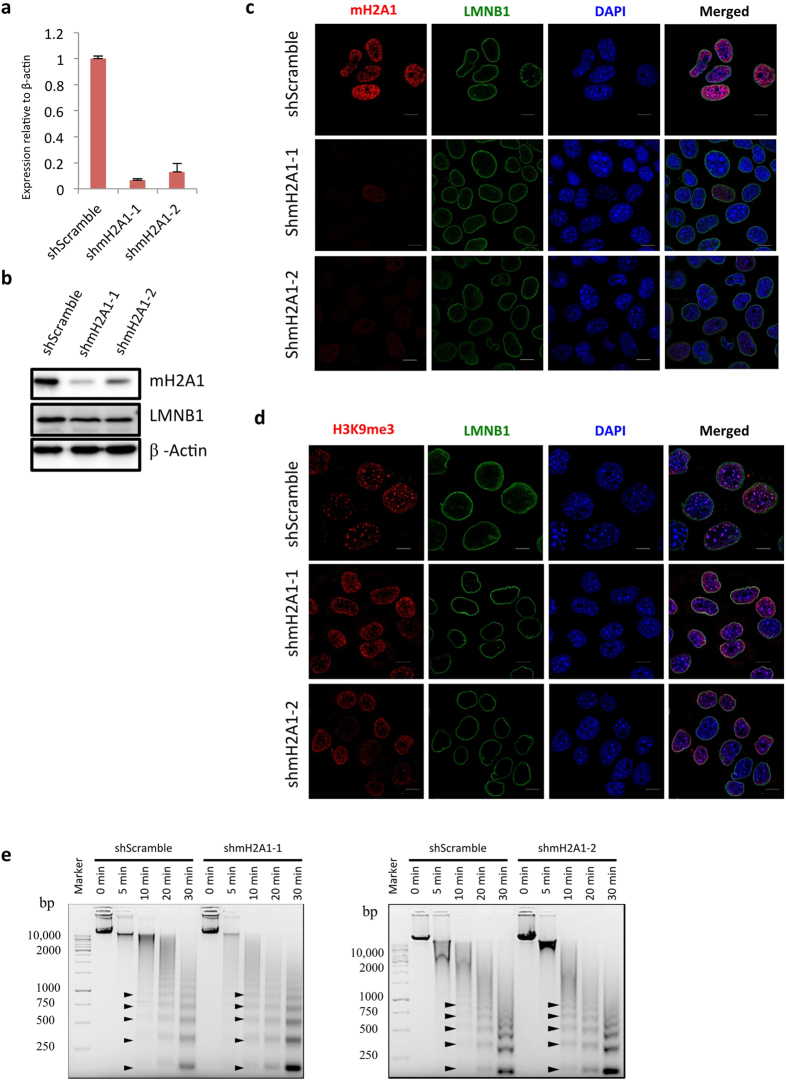
Knockdown of macroH2A1 changes chromatin architecture in mouse hepatocytes. Knockdown efficiency of macroH2A1 in AML-12 cells as tested by qRT-PCR (**a**), western blots (**b**) and immunofluorescence (**c**). Relative expression level of the scramble control was set as 1 in qRT-PCR experiments. (**d**) Sub-nuclear localization of the heterochromatin marker H3K9me3 upon macroH2A1 knockdown. Confocal images show localization of H3K9me3 (red), LNMB1 (green) and DAPI (blue), Scale = 10 μm. (**e**) MNase assay for macroH2A1 RNAi and negative control cells, Black arrows indicate nucleosome ladders from MNase digestion.

To reveal potential roles of macroH2A1 in chromatin organization, we silenced the expression of macroH2A1 by RNA interference in the AML12 cells. The knockdown efficiency of two independent shRNAs was confirmed by quantitative RT-PCR (Fig. 4a), western blot (Fig. 4b), and Immunofluorescence (Fig. 4c). We then checked cellular distribution of histone modifications H3K9me3, the marker of heterochromatin. Typical H3K9me3 foci can be clearly seen in the controls (top panel of Fig. 4d). However, upon macroH2A1 knockdown, the H3K9me3 foci were largely released and spread over the nucleus (Fig. 4d). Moreover, when macroH2A1 was down regulated, global decondensation of chromatin structure can be detected with Micrococcal nuclease (MNase) assay (Fig. 4e), consistent with the results observed in melanoma cells^20^. For example, we observed prominent nucleosome ladders after 10 min of MNase digestion at knockdown of macroH2A1 but not in the scramble control sample (Fig. 4e). These data indicated that macroH2A1 is required for stabilizing the chromatin architecture in mouse liver cells.

### Down-regulation of LMNB1 results in the redistribution of macroH2A1

**Figure 5:**
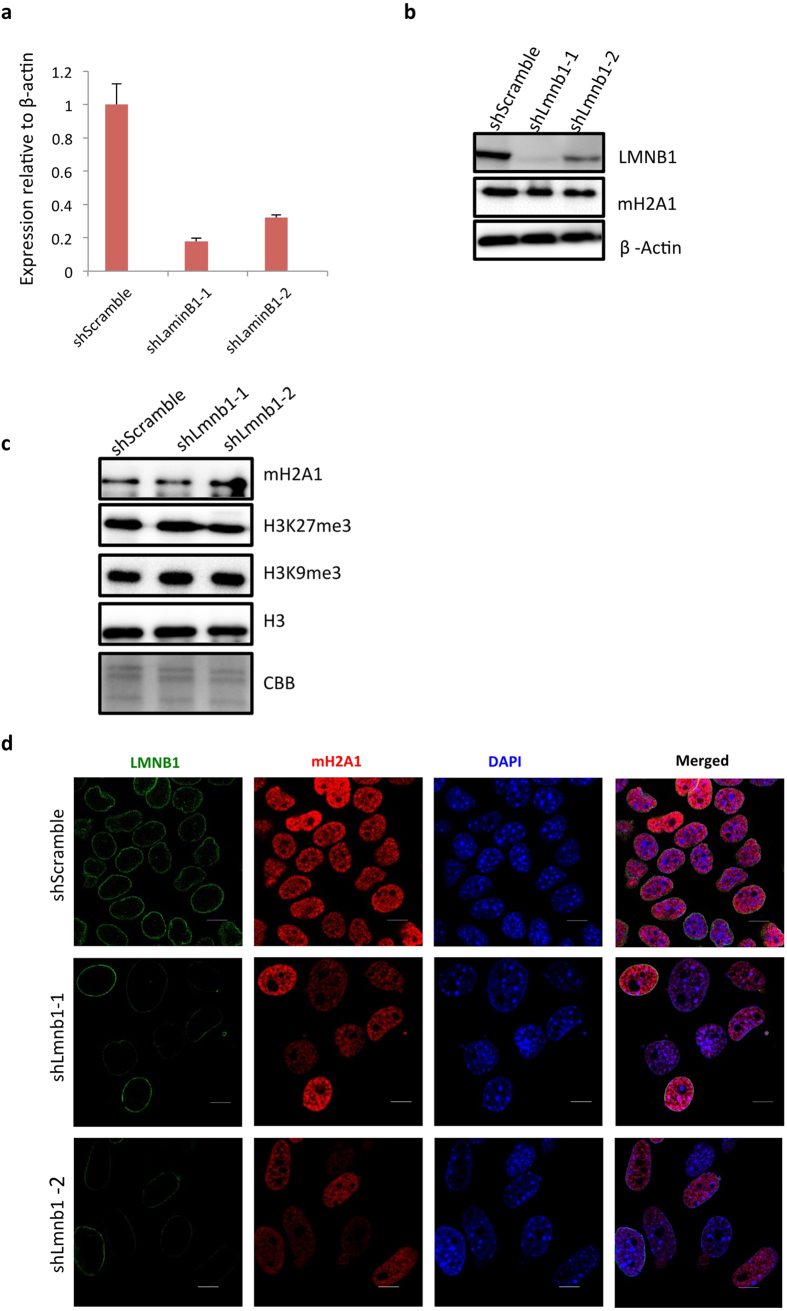
Down regulation of LMNB1 results in redistribution of macroH2A1 in mouse hepatocytes. (**a**) Knockdown efficiency as tested by qRT-PCR. Relative expression level of the scramble control was set as 1. (**b**) Total protein levels as shown by western blots using whole cell extract. B-Actin was used as loading control. (**c**) MacroH2A1, histone modifications (H3K27me3 and H3K9me3) and total histone H3 detected by western blots using histone extracts, Both H3 western blot and Comassie Blue Brilliant staining (CBB) are as loading controls. (**d**) Immunofluorescence of LMNB1 (green) and macroH2A1 (red) upon LMNB1 knockdown. Scale = 10 μm.

To further elucidate the relationship between LMNB1 and macroH2A1, we knock-downed the expression of LMNB1 in AML12 cells (Fig. 5a). The total levels of macroH2A1 were largely unchanged upon LMNB1 knockdown, as shown by western blots based on whole cell lysate (Fig. 5b) and histone extract (Fig. 5c). However, IF experiments showed that macroH2A1 signals became weaker in the cells of which LMNB1 had been silenced (Fig. 5d). These data suggested that loss of LMNB1 results in redistribution of macroH2A1 in the nucleus.

## Discussion

Protein networks associated with lamins are very difficult to be analyzed by conventional affinity complex purification, mainly due to the insolubility of lamina. In BioID experiments, the BirA* fusion protein biotinylates proximate proteins in a relatively natural cellular environment, and then the biotinylated proteins can be isolated by streptavidin beads, thus the issue of insolubility can be overcome^18^. Moreover, biotinylation is a covalent modification and the biotin-avidin interactions are nearly covalent, permitting complete cell lysing and stringent washes by using strong detergents, such as 2% SDS. These advantages make BioID an ideal tool to analyze insoluble and membrane-associated proteins such as lamins. This method was applied to analyze proteins associated with lamin A^18^. In addition, BioID has been used to screen other “difficult complexes” such as cell junction complexes^21^,^22^, centrosomes^23^ and nuclear pore complexes^24^. Most of published BioID-based studies were conducted on “easy-to-transfect” cell lines such as Hela and 293T. In this report, we engineered the BirA* element into lentiviral vectors. This virus based BioID protocol can be applied to most of cell types, including normal or primary cells that are closer to real physiological conditions.

Using this virus-based BioID assay, we identified 182 LMNB1-associated proteins in mouse liver cells. Based on the BioGRID database that integrates the data of published literatures^25^, there are 67 proteins associate with LMNB1 in human (64 proteins) or mouse (5 proteins), 7 (10%) of which are also identified in this report, and 175 associated proteins were firstly identified here. Besides known components of nuclear envelope, many proteins are associated with chromosome organization. These included some known proteins that function in chromatin-lamina tethering, such as LMNA, LBR, LAP2, MAN1^9^,^26^. It can be speculated that some novel candidates in the list could direct chromatin-lamina interaction, which call for further investigations. Furthermore, consistent with previous observation that RNA splicing factors localized with lamins^27^, there are many RNA splicing factors in the list, such as PTBP1, although their detailed roles in nuclear periphery are to be uncovered. Taken together, the proteomics data reported in this study may provide candidates for further studies regarding biological roles of lamina.

Genome-wide molecular mapping studies have identified thousands of LADs in Drosophila, human and mouse genomes^6^,^28^. In this report, we constructed the first map of LMNB1-chromatin association in mouse hepatocytes, and identified 1317 LADs. We extensively analyzed the epigenomic signatures of these LADs, and found that LADs are associated with macroH2A1-containing chromatin. Our paper thus provides useful resources to reveal the lamina-dependent chromatin organization in liver cells.

MacroH2A1 had long been recognized as a hallmark of facultative heterochromatin, and was originally linked to inactive X chromosome^29^,^30^. Further studies indicated that macroH2A1 histones are associated with repressive chromatin genome-widely^31^,^32^. Importantly, macroH2A1 contributed to the establishment and the maintenance of epigenetic states of differentiated cells and was a barrier for cellular reprogramming^33^,^34^,^35^. Besides, the levels of macroH2A1 are reduced in many cancer types^36^,^37^. In this report, we demonstrated that macroH2A1 is associated with nuclear lamina and required to maintain chromatin structure. These data suggested that macroH2A1 could regulate lamina-dependent higher-order chromatin in development and diseases, which await for further exploring.

Previous studies indicated that LMNB1 is required to maintain the organization of chromatin in interphase nuclei^38^, and depletion of LMNB1 lead to de-condensation of chromosome territories and affect chromosome positioning^39^,^40^. We further demonstrate that macroH2A1 associated with LMNB1, and down-regulation of LMNB1 resulted in redistribution of macroH2A1 protein, consistent with a previous report showing redistribution of H3K27me3^39^. These data suggested that LMNB1 is required to maintain global chromatin structure, at least partially by regulating repressive histone marks including H3K27me3 and macroH2A1.

In summary, using virus-based BioID techniques, we generated the first proteome associated with B-type lamins in mouse liver cells, and identified 182 candidate proteins. We constructed the genome-wide map of Lamina-chromatin association in mouse hepatocytes, and identified 1,317 LADs. Furthermore, we demonstrated that both LMNB1 and LMNB1-associated chromatin are associated with macroH2A, and found that loss of LMNB1 results redistribution of macroH2A1 histones. Finally, macroH2A1 contributes to the maintenance of the chromatin architecture in mouse hepatocytes. Taken together, we provided proteomics and genomics data for studies regarding the biological function and mechanisms of the nuclear lamina, and provided evidences for the lamina-macroH2A1 association as well as its biological roles to maintain genome organization.

## Materials and Methods

### Plasmid construct for BioID

The full-length mouse *Lmnb1* cDNA was amplified with primers: GCGC**AGATCT**ATGGCGACCGCGACCCCCGTGCA and GCGC**GGCGCGCC**TCACATAATGGCACAGCTTTTATTC. The Lmnb1 cDNA was cloned into the pcDNA3.1mycBioID plasmid, which was created by Roux *et al.*^18^ and was obtained from Addgene. To construct the lentivirus-based vectors, MycBirA* or MycBirA*-Lmnb1 fragments were amplified and cloned into the lentiviral plasmid. To construct the MycBirA*-macroH2A1 vector, human *macroH2A1* cDNA was amplified using primers: GGCC**GCGGCCGC**ATGTCGAGCCGCGGTGG, and GGCC**GAATTC**CTAG TTGGCGTCCAGCTTGG. The *macroH2A1* cDNA was cloned into pcDNA3.1mycBioID vector. All constructs were verified by DNA sequencing.

### BioID assay

For virus-based BioID, AML12 cells (ATCC, CRL-2254™) cells were infected with lentivirus particles of Lv-MycBirA*-Lmnb1. Twenty-four hours after infection, 4 μM of Dox +) was added, and 48 hours after infection, the cells were treated with 50 μM of biotin for 8 hours. Affinity capture of biotinylated proteins was performed as described^18^, except that we used High Capacity Streptavidin Agrose beads (Thermo Scientific) instead of magnetic beads. Same amount (1 × 10^8^) of Dox (+) and Dox (−) samples were used for one BioID experiment. Eluted biotinylated proteins were separated on SDS-PAGE and visualized by silver staining. For western blot analysis, 10% of eluted product was loaded for one blot.

### Mass Spectrometry (MS) and data analysis

To prepare samples for MS analysis, eluted biotinylated proteins were separated by SDS-PAGE, and visualized by coomassie staining. The gel lanes were equally excised and divided into 3 parts, each of which was used for one MS analysis. The LC-MS/MS experiments were performed on a Nano UPLC system (Waters Corporation) connected to a quadrupole-Orbitrap mass spectrometer (Q-Exactive) (ThermoFisher Scientific) equipped with an online nano-electrospray ion source.

The mass spectra were searched using the Mascot Daemon software (Version 2.3.0) based on the Mascot algorithm. The UniProtKB/Swiss-Prot database (Release 2014_02_19, with 16648 entries) was used to search. To reduce false positive identification results, a decoy database containing the reverse sequences was appended to the database. The searching parameters were set up as follows: full trypsin (KR) cleavage with four missed cleavage was considered. Oxidation on methionine and Biotinylation on lysine were set as variable modifications. The peptide mass tolerance was 10 ppm and the fragment ion tolerance was 0.05 Da. Filter parameter: Significance threshold p < 0.05, Ions score or expect cut of 13 and choose show percolator scores; use the percolator algorithm to control peptide level false discovery rates (FDR) lower than 1%.

MS analyses of BirA*-Lmnb1and BirA* BioID products were performed on three biological replicates in AML12 cells. Proteins that appear at least twice in the three replicates were considered as positive hits. Keratins and other epidermal proteins, proteases and endogenously biotinylated carboxylases were discarded for further analysis as described^21^. To compare relative abundance of proteins among different mass spectrometry analyses, PSM (peptide spectral match) values for individual proteins were normalized with the total PSMs for each run^21^, and normalized PSMs of three replicates were averaged. Protein hits only identified in BirA*-lmnb1 BioID, and hits whose PSMs in BirA*-lmnb1 BioID were 2-fold greater than those of BirA* alone were considered as candidates of LNMB1 associated proteins. The protein list was used for Gene Ontology (GO) analysis via the DAVID bioinformatics tools^41^.

### DamID and data analysis

DamID experiments were performed as described^6^ in AML-12 cells. Dam-only and Dam-LMNB1 samples were labeled with Cy3 and Cy5 respectively, and were hybridized to mouse CGH whole genome tiling arrays (Agilent Technologies). Arrays were scanned to obtain probe intensity and log2 ratios of Dam-LMNB1/Dam-only. The experiments were performed for four biological replicates. The DamID microarray data were deposited in the GEO database with Series entry number GSE73703.

All data analyses were performed using R programming language. To detect Lamina associated chromatin domains (LADs), DamID data were first normalized and smoothed. We smoothed the probe signals using a moving average approach with a window size of 100 k bps. The smoothing procedure takes into consideration of the spatial correlation of signals and increases power of LADs detection. Next at each probe, we converted the smoothed signals from four replicates into one t-statistics using the mean divided by standard deviation. We then determined a t-statistics threshold for calling LADs based on probe level false discovery rate (FDR), using the left part of the distribution as null (Figure S2). Under FDR 0.01, the threshold is −3. We defined LADs as regions with consecutive probes with t-statistics greater than the threshold. We also required minimum length of LADs to be 100 k bps and containing at least 50 probes. LADs with distances <5 kb were merged into one LAD.

To explore epigenomic features of LADs, chromatin markers H3K9me2, CTCF, H3K27me3, H3K4me3 and macroH2A were plotted against LAD boundaries (±200 kb). Firstly, we combined left and mirrored right boundaries, and converted all features to coordinates relative to the nearest boundary. Next, we constructed continuous 5 kb sliding windows from outside to inside to calculate the averaged signal according to the position of each LAD. Finally, we profiled the averaged data, and each plot reflects the average signal across all boundaries. Datasets of liver cell were obtained from the Gene Expression Omnibus (GEO) database: H3K9me2 (GSE28581), macroH2A (GSM469459). CTCF (GSM918715), H3K27me3 (GSM1000150) and H3K4Me3 (GSM769014).

### RNA interference

For the RNAi experiments, shRNA clones were picked up from The RNAi Consortium (TRC) libraries: Lmnb1 (TRCN0000091903 and TRCN0000091904); macroH2A1 (TRCN0000097040 and TRCN0000097040). As a control, a 21-mer scrambled sequence, ACTCGACACTATAGTATCTCA, was cloned into pLKO.1-TRC cloning vector. To produce shRNA lentivirus, 293T cells were transfected with 1 μg shRNA vector, 0.75 μg pspax2 plasmid and 0.25 μg pMD2.G plasmid with 5 μl FuGENE 6 in 200 μl Opti-MEM per well. For RNAi experiments, 20,000 of AML12 cells were infected with 100 μl of virus-containing supernatant in a well of six-well-plates. Medium was changed 12 hours after transfection, and then 1 μg/ml of puromycin was added for cell selection for 48 hours or longer.

### Immunofluorescence

Immunofluorescence was performed as described^42^ with minor modifications. Briefly, after washing, fixing and permealizing, cells were incubated overnight at 4 °C with primary antibodies: goat polyclonal Lamin B1 (sc-6216, Santa Cruz Biotechnology), mouse monoclonal Myc (60003-2-Ig, Proteintech), rabbit monoclonal MacroH2A1 (ab183041, Abcam), rabbit polyclonal H3K9me3 (A2360, Proteintech), rabbit monoclonal H3K27me3 (9733, CST) and rabbit monoclonal H3K4me3 (9751, CST). After incubation, cells were washed three times with blocking buffer (4% BSA in 1XPBS) and incubated two hours with secondary antibodies: Alexa Flour488 donkey anti-goat, Dylight 594 donkey anti-mouse, Dylight 594 donkey anti-rabbit (all from Jackson immunoResearch Lab). For biotinylated protein staining, cells were incubated with fluorescent Dye 647-Streptavidin (U0297, Abnova) for 2 hour at RT. After incubation with secondary antibodies or streptavidin, coverslips were washed three times and counterstained with 0.5 mg/ml DAPI (Beyotime Biotechnology) for 5 minutes at RT. Then coverslips were mounted in vectashield mounting medium (H-1000, Vector, Burlingame) and sealed on slides.

### Imaging with confocal microscope and Structured Illumination Microscopy (SIM)

Confocal images were taken with a confocal laser scanning microscopy (Leica, SP5), using parameters as followings: Apochromat 63 × 1.4 NA oil immersion objective lens; zoom = 3; Laser lines 405 nm, 488 nm, 594 nm, and 647 nm to excite DAPI, AlexaFluor 488, Dylight 594 and Dylight 647, respectively. Digital images were analyzed using the Leica software (LAS AF Lite).

For SIM imaging, immunofluorescence preparations were observed on an Eclipse Ti inverted microscope equipped with a Nikon Plan Apo 3100 TIRF objective (NA 1.49, oil immersion; Nikon, NIS-elements AR). For imaging three lasers SIM405, SIM488 and SIM 594, z-step size was set to 0.20 um; for each focal plane, 15 images (5 phases, 3 angles) were captured with the NIS-Elements software. SIM images were reconstructed, acquired and analyzed using the N-SIM module of the NIS-Elements Advanced Research 3.0 software. The parameters of reconstruction are as follows: Illumination modulation contrast, auto; High resolution noise suppression, 1.00; Out of focus blur suppression 0.05.

### Micrococcal nuclease (MNase) assays

MNase assay was conducted as described^20^ with some modification. Briefly, cells were counted and evenly aliquoted (2.5 × 10^6^ cells per assay), and each sample was treated with 1/150 unit of micrococcal nuclease for 0, 5, 10, 20 or 30 min at 37 °C. DNA samples was purified by equal volume of phenol/chloroform. Equal amounts of DNA (800 ng/lane) were resolved on 1.5% agarose gel.

### Western Blot

Protein samples were separated on SDS-PAGE and transferred to polyvinylidene difluoride membranes, and probed with primary antibodies: anti-Lamin B1 (sc-6216, Santa Cruz Biotechnology), anti-macroH2A1 (ab183041, Abcam), anti-LAP2 (14651-1-AP, Proteintech), anti-EMD (10351-1-AP, Proteintech), anti-MAN1 (sc-50458, Santa Cruz Biotechnology), anti-Lamin A/C (sc-6215, Santa Cruz Biotechnology); anti H3 (ab1791, abcam), anti H3K9me3 (A2360, Proteintech), anti H3K27me3 (9733, CST), anti-b-Actin (AGM11086, AOGMA) and anti-GAPDH (AGM12183, AOGMA). Then bound primary antibodies were detected with AffiniPure peroxidase–conjugated 2^nd^ antibodies (Jackson immunoResearch Lab). The blots were developed with ECL Plus Western Blotting Substrate (32132, Thermo Scientific), and imaged by the FluorChem M System (ProteinSimple, Santa Clara, CA, US).

### qRT-PCR

RNA was isolated using TRIZOL (Life Technologies). cDNA was generated by FastQuant RT Kit (Tiangen, Beijing, China). Quantitative PCR was performed with 2× PCR SYBR®Green master mix (Roche) on the Lightcycle96 real-time PCR system (Roche). Primers were as follows: macroH2A forward, 5′-CTGCCTGCCAAGTTTGTGAT-3′; macroH2A reverse, 5′-CAATGGAT GGGAAGGCGATG-3′; Lmnb1 forward, 5′-CAACTGACCTCATCTGGA AGAAC-3′; Lmnb1 reverse, 5′-CTTGAAGACTGTGCTTCTCTGAG-3. B-Actin forward: 5′-GGTCATCACTATTGGCAACG; B-Actin reverse: 5′-ACGGATGTC AACGTCACACT.

## Additional Information

**How to cite this article**: Fu, Y. *et al.* MacroH2A1 associates with nuclear lamina and maintains chromatin architecture in mouse liver cells. *Sci. Rep.* **5**, 17186; doi: 10.1038/srep17186 (2015).

## Supplementary Material

Supplementary Information
