# Supplementary material for: MacroH2A1 associates with nuclear lamina and maintains chromatin architecture in mouse liver cells
Source: Sci Rep. 2015 Nov 25;5:17186. doi: 10.1038/srep17186 (PMC4658601; doi:10.1038/srep17186)
Supplement: Supplementary Information [file srep17186-s1.pdf]

Supplementary Information

MacroH2A1 associates with nuclear lamina and maintains chromatin architecture in mouse liver cells

Yuhua Fu[1], Pin Lv[1], Guoquan Yan[1-2], Hui Fan[1], Lu Cheng[1], Feng Zhang[3], Yongjun Dang[1], Hao Wu[4] and Bo Wen[1,3*]

[1] Key Laboratory of Molecular Medicine of Ministry of Education and Institutes of Biomedical Sciences, Shanghai Medical College, Fudan University, Shanghai 200032, China; [2] Department of Chemistry, Fudan University, Shanghai, 200433; [3]State Key Laboratory of Genetic Engineering and Collaborative Innovation Center for Genetics and Development, School of Life Sciences, Fudan University, Shanghai 200438, China; [4]Department of Biostatistics and Bioinformatics, Emory University, Atlanta, GA 30322, USA.

[*]Author for correspondence (bowen75@fudan.edu.cn)

A

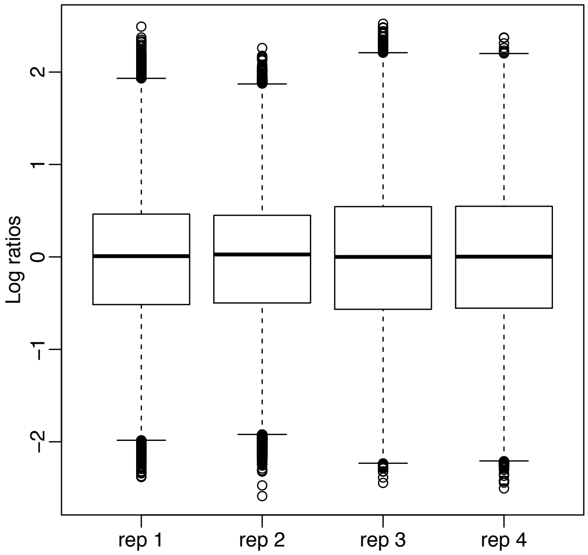

B

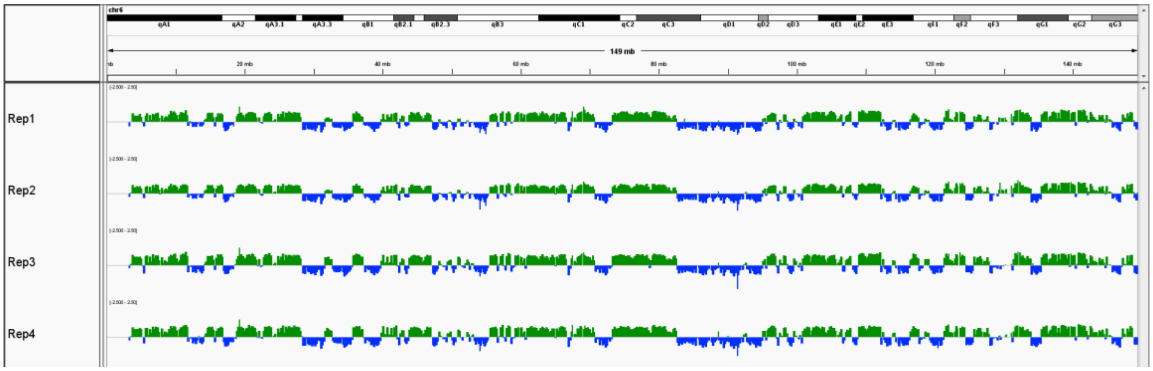

Figure S1. **Reproducibility of biological replicates of LMNB1 DamID in AML12 cells.** (A) Boxplot of LMNB1 signals (log2 rations of Dam-LMNB1/Dam-only) from replicates. Average correlation of data from the four replicates is 0.85. (B) LMNB1 signals across genome, as exemplified by chromes 6, show almost identical distributions among replicates. These results indicate a highly reproducibility of these experiments.

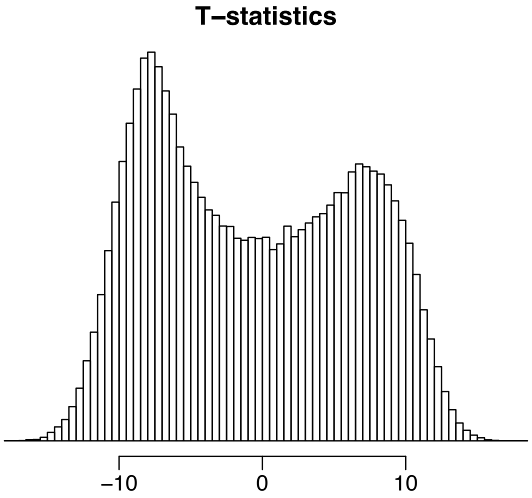

Figure S2. Histogram of probe level t-statistics from smoothed signals. Histogram of the resulting test statistics shows a clear bimodal distribution. Two parts of the distribution represent probes from Lamin B1 associated chromatin domains (LADs) and non-LADs.

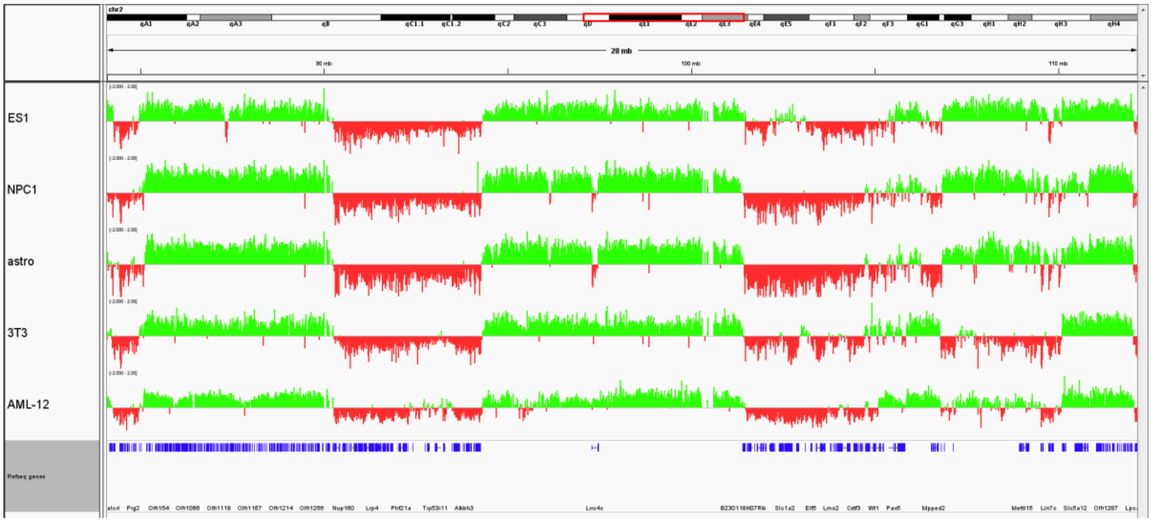

Figure S3. Comparison of LMNB1-chromation association maps among cell types. Showing is LMNB1 signals of embryonic stem cells (ES), neural progenitor cells (NPC), Astrocytes (Astro), 3T3 cells and AML12 cells, on a 28 Mb region of chromosome 2.

Table S1. Proteins associated with Lamin B1 as identified by BioID

Gene	Protein ID	BirA*-Lmnb1 BioID # of matches Rep1	Rep2	Rep3	PSM	BirA* BioID # of matches Rep1	Rep2	Rep3	PSM
Actn1	Q7TPR4	388	372	386	278.1	10	12	35	49.1
Actn2	Q9JI91	0	113	125	56.9	0	0	0	0
Actn4	P57780	387	380	383	279.1	0	0	0	0
Ahctf1	Q8CJF7	12	13	12	9.0	0	0	0	0
Ankfy1	Q810B6	1	1	1	0.7	0	0	0	0
Anp32b	Q9EST5	0	3	2	1.2	0	0	0	0
Arhgdia	Q99PT1	1	3	2	1.4	0	0	0	0
Arhgef17	Q80U35	2	4	3	2.2	0	0	0	0
Bzw1	Q9CQC6	2	1	1	1.0	0	0	0	0
Capza1	P47753	29	22	27	19.0	1	1	1	2.7
Ccar1	Q8CH18	0	2	2	1.0	0	0	0	0
Ccdc47	Q9D024	1	0	1	0.5	0	0	0	0
Ccdc50	Q810U5	1	1	1	0.7	0	0	0	0
Ccnd1	P25322	1	1	0	0.5	0	0	0	0
Cdc37	Q61081	1	1	2	1.0	0	0	0	0
Cdc42bpa	Q3UU96	1	3	0	1.0	0	0	0	0
Cdc42bpb	Q7TT50	1	2	0	0.7	0	0	0	0
Cdc42ep1	Q91W92	2	1	0	0.7	0	0	0	0
Cdc5l	Q6A068	1	1	0	0.5	0	0	0	0
Cdk18	Q04899	0	1	1	0.5	0	0	0	0
Cdk4	P30285	1	2	2	1.2	0	0	0	0
Chchd3	Q9CRB9	1	1	3	1.2	0	0	0	0
Chd4	Q6PDQ2	3	2	2	1.7	0	0	0	0
Chordc1	Q9D1P4	1	1	1	0.7	0	0	0	0
Chtop	Q9CY57	2	1	4	1.7	0	0	0	0
Cops4	O88544	1	1	0	0.5	0	0	0	0
Cpsf3l	Q9CWS4	0	2	1	0.7	0	0	0	0
Csrp1	P97315	1	1	2	1.0	0	0	0	0
Ctnna1	P26231	5	2	5	2.9	0	0	0	0
Cystm1	Q8K353	1	0	1	0.5	0	0	0	0
Dctn1	O08788	1	1	1	0.7	0	0	0	0
Ddx1	Q91VR5	3	5	4	2.9	0	0	0	0
Ddx50	Q99MJ9	1	5	4	2.4	0	0	0	0
Ddx6	P54823	1	1	0	0.5	0	0	0	0
Dhx15	O35286	2	2	1	1.2	0	0	0	0
Dnajb6	O54946	0	1	1	0.5	0	0	0	0
Eef2	P58252	19	21	19	14.3	1	3	3	6.0
Eif1ax	Q8BMJ3	1	1	1	0.7	0	0	0	0
Eif2s1	Q6ZWX6	1	2	1	1.0	0	0	0	0
Eif3c	Q8R1B4	3	6	3	2.9	0	0	0	0
Eif3d	O70194	5	5	4	3.4	0	0	0	0
Eif3e	P60229	1	1	2	1.0	0	0	0	0

Eif3g	Q9Z1D1	3	3	2	1.9	0	0	0	0
Eif3h	Q91WK2	1	2	1	1.0	0	0	0	0
Eif3i	Q9QZD9	2	5	2	2.2	0	0	0	0
Eif4a3	Q91VC3	5	6	8	4.6	0	0	0	0
Eif4b	Q8BGD9	1	1	1	0.7	0	0	0	0
Eif5a2	Q8BGY2	1	0	1	0.5	0	0	0	0
Emd	O08579	5	8	6	4.6	0	0	0	0
Etf1	Q8BWY3	0	1	1	0.5	0	0	0	0
Ezr	P26040	20	20	19	14.3	0	5	3	6.6
Faf2	Q3TDN2	0	1	1	0.5	0	0	0	0
Fkbp5	Q64378	5	8	6	4.6	1	0	1	1.9
Flnb	Q80X90	22	22	22	16.0	2	2	5	7.8
Fmnl2	A2APV2	6	6	5	4.1	0	0	0	0
Fus	P56959	3	2	2	1.7	0	0	0	0
G3bp1	P97855	3	3	0	1.5	0	0	0	0
Gdi2	Q61598	2	2	2	1.5	0	0	0	0
Gtf2i	Q9ESZ8	1	0	2	0.7	0	0	0	0
H2afx	P27661	3	0	3	1.5	0	0	0	0
H2afy	Q9QZQ8	1	1	1	0.7	0	0	0	0
H2afz	P0C0S6	3	3	3	2.2	0	0	0	0
Hist1h1d	P43277	3	0	6	2.2	0	0	0	0
Hist1h1e	P43274	3	4	6	3.1	0	0	0	0
Hmgb1	P63158	0	1	1	0.5	0	0	0	0
Hmgb2	P30681	3	1	1	1.2	0	0	0	0
Hnrnpa0	Q9CX86	1	0	2	0.7	0	0	0	0
Hnrnpa1	P49312	5	6	5	3.9	0	0	0	0
Hnrnpa2b1	O88569	8	6	5	4.6	0	0	0	0
Hnrnpa3	Q8BG05	4	5	3	2.9	0	0	0	0
Hnrnpab	Q99020	2	0	1	0.7	0	0	0	0
Hnrnpc	Q9Z204	1	2	1	1.0	0	0	0	0
Hnrnph1	O35737	6	9	8	5.6	0	0	0	0
Hnrnpk	P61979	9	11	10	7.3	0	1	1	1.6
Hnrnpul2	Q00PI9	2	3	1	1.5	0	0	0	0
Hp1bp3	Q3TEA8	1	0	2	0.7	0	0	0	0
Hspa4	Q61316	3	2	3	1.9	0	0	0	0
Ilk	O55222	2	1	3	1.5	0	0	0	0
Ipo7	Q9EPL8	2	0	1	0.7	0	0	0	0
Ipo9	Q91YE6	0	1	4	1.2	0	0	0	0
Khdrbs1	Q60749	1	3	1	1.2	0	0	0	0
Kiaa1671	Q8BRV5	1	3	2	1.4	0	0	0	0
Kpnb1	P70168	4	7	5	3.9	0	0	0	0
Kpna6	O35345	2	2	2	1.5	0	0	0	0
Lad1	P57016	11	13	10	8.2	1	0	1	1.9
Lbr	Q3U9G9	25	30	25	19.4	0	2	2	3.3
Lemd3	Q9WU40	10	7	9	6.3	0	0	0	0
Lima1	Q9ERG0	33	27	37	23.5	0	0	0	0

Lmna	P48678	137	138	129	98.1	12	15	11	33.6
Lmnb1	P14733	144	147	150	107.0	0	0	0	0
Lmnb2	P21619	37	46	40	29.8	0	0	0	0
Lrrc16a	Q6EDY6	1	1	0	0.5	0	0	0	0
Lrrfip1	Q3UZ39	2	3	5	2.4	0	0	0	0
Lrrfip2	Q91WK0	12	14	15	9.9	0	0	0	0
Ltbp3	Q61810	0	1	1	0.5	0	0	0	0
Luzp1	Q8R4U7	2	2	2	1.5	0	0	0	0
Mcm3	P25206	3	3	3	2.2	0	0	0	0
Mcm6	P97311	4	7	5	3.9	0	0	0	0
Misp	Q9D279	0	1	1	0.5	0	0	0	0
Nap1l4	Q78ZA7	1	1	1	0.7	0	0	0	0
Ncl	P09405	4	6	4	3.4	0	0	0	0
Ndca	Q60817	2	0	2	1.0	0	0	0	0
Nup155	Q99P88	0	1	2	0.7	0	0	0	0
Nup50	Q9JIH2	1	3	2	1.4	0	0	0	0
Nup93	Q8BJ71	3	2	3	1.9	0	0	0	0
Nup98	Q6PFD9	1	3	2	1.4	0	0	0	0
Pa2g4	P50580	0	1	1	0.5	0	0	0	0
Pard6b	Q9JK83	1	0	1	0.5	0	0	0	0
Pdcd6ip	Q9WU78	2	3	5	2.4	0	0	0	0
Pdzd11	Q9CZG9	4	3	3	2.4	0	0	0	0
Pgrmc1	O55022	2	1	3	1.5	0	0	0	0
Pgrmc2	Q80UU9	3	3	1	1.7	0	0	0	0
Phldb2	Q8K1N2	6	9	13	6.8	1	0	2	2.7
Plekha7	Q3UIL6	26	23	22	17.3	0	0	0	0
Poldip3	Q8BG81	1	0	1	0.5	0	0	0	0
Pom121	Q8K3Z9	2	2	1	1.2	0	0	0	0
Ptbp1	P17225	3	2	2	1.7	0	0	0	0
Ptbp3	Q8BHD7	1	1	1	0.7	0	0	0	0
Ptrf	O54724	3	1	2	1.5	0	0	0	0
Rab11b	P46638	0	2	1	0.7	0	0	0	0
Rab21	P35282	1	1	0	0.5	0	0	0	0
Rab38	Q8QZZ8	3	1	2	1.5	0	0	0	0
Rab7a	P51150	1	1	1	0.7	0	0	0	0
Rac1	P63001	2	0	2	1.0	0	0	0	0
Ralb	Q9JIW9	1	2	1	1.0	0	0	0	0
Rangap1	P46061	1	1	1	0.7	0	0	0	0
Rap1b	Q99JI6	2	3	2	1.7	0	0	0	0
Rbm7	Q9CQT2	1	1	1	0.7	0	0	0	0
Rbmxl1	Q91VM5	3	0	3	1.5	0	0	0	0
Rhoa	Q9QUI0	0	1	1	0.5	0	0	0	0
Rhog	P84096	2	1	1	1.0	0	0	0	0
Rpa2	Q62193	1	1	0	0.5	0	0	0	0
Rras	P10833	4	4	4	2.9	0	0	0	0
Rras2	P62071	4	3	4	2.7	0	0	0	0

Scc1	Q5SXY1	21	16	17	13.1	0	0	0	0
Scc1l	Q2KN98	25	17	24	16.0	0	0	0	0
Sec61a1	P61620	6	6	5	4.1	0	0	0	0
Set	Q9EQU5	2	1	3	1.5	0	0	0	0
Sf3a1	Q8K4Z5	1	2	2	1.2	0	0	0	0
Sfpq	Q8VIJ6	11	9	9	7.1	0	0	0	0
Shkbp1	Q6P7W2	1	1	1	0.7	0	0	0	0
Sipa1	P46062	6	6	6	4.4	0	0	0	0
Sipa1l1	Q8C0T5	8	7	7	5.3	0	0	0	0
Slc2a1	P17809	1	1	1	0.7	0	0	0	0
Slc30a7	Q9JKN1	1	2	1	1.0	0	0	0	0
Smarca5	Q91ZW3	0	4	3	1.7	0	0	0	0
Smc3	Q9CW03	6	3	7	3.9	0	0	0	0
Smc4	Q8CG47	1	1	3	1.2	0	0	0	0
Snrnp200	Q6P4T2	2	2	2	1.5	0	0	0	0
Sptan1	P16546	68	67	73	50.5	0	0	0	0
Srprb	P47758	2	1	1	1.0	0	0	0	0
Srrm2	Q8BTI8	0	3	3	1.4	0	0	0	0
Srsf1	Q6PDM2	1	1	1	0.7	0	0	0	0
Srsf2	Q62093	1	1	1	0.7	0	0	0	0
Stub1	Q9WUD1	0	1	1	0.5	0	0	0	0
Sun2	Q8BJS4	0	1	1	0.5	0	0	0	0
Supt5h	O55201	2	2	2	1.5	0	0	0	0
Tjp2	Q9Z0U1	34	48	38	29.1	3	4	4	9.7
Tm9sf3	Q9ET30	1	1	1	0.7	0	0	0	0
Tmed10	Q9D1D4	1	2	1	1.0	0	0	0	0
Tmed2	Q9R0Q3	1	1	1	0.7	0	0	0	0
Tmem106b	Q80X71	0	1	1	0.5	0	0	0	0
Tmem43	Q9DBS1	2	1	2	1.2	0	0	0	0
Tmem63a	Q91YT8	1	0	1	0.5	0	0	0	0
Tmpo	Q61029	59	44	49	37.0	2	4	4	8.6
Tor1aip1	Q921T2	3	2	5	2.4	0	0	0	0
Tpr	F6ZDS4	1	0	2	0.7	0	0	0	0
Tra2b	P62996	1	0	1	0.5	0	0	0	0
Traf4	Q61382	8	6	5	4.6	0	0	0	0
Trim28	Q62318	0	1	3	1.0	0	0	0	0
U2af2	P26369	1	0	1	0.5	0	0	0	0
Uba1	Q02053	6	7	5	4.4	0	0	0	0
Uso1	Q9Z1Z0	0	5	1	1.4	0	0	0	0
Utp11l	Q9CZJ1	1	0	2	0.7	0	0	0	0
Utp18	Q5SSI6	0	1	1	0.5	0	0	0	0
Vapb	Q9QY76	1	3	1	1.2	0	0	0	0
Vasp	P70460	1	1	4	1.4	0	0	0	0
Wdr82	Q8BFQ4	1	1	1	0.7	0	0	0	0
Ybx1	P62960	3	0	2	1.2	0	0	0	0
Znf185	Q62394	4	8	5	4.1	0	0	0	0

Znf512	Q69Z99	0	1	2	0.7	0	0	0	0
Znf622	Q91VY9	0	1	1	0.5	0	0	0	0

Table S2. Coordinates (mm9) of LADs in mouse liver cells as identified by DamID

Chr	Start	End
chr1	3590700	4698168
chr1	4916174	6149247
chr1	6260726	9504039
chr1	11145879	13028380
chr1	13761319	14445819
chr1	14522876	14866093
chr1	15238264	15768206
chr1	16769850	20054293
chr1	21298377	21436486
chr1	21671987	23126335
chr1	23439074	23859358
chr1	24147814	24686290
chr1	24810706	27601891
chr1	27658933	30773025
chr1	31256331	33454991
chr1	34509378	34829277
chr1	34970167	35824149
chr1	38259145	39383729
chr1	40637524	41582923
chr1	41664953	42912447
chr1	42982097	43124690
chr1	44224734	45823809
chr1	46983127	50964213
chr1	52044304	52146469
chr1	52913599	53719493
chr1	53954474	54576405
chr1	55237047	56562121
chr1	57031280	57403351
chr1	60963909	61284650
chr1	61343484	62712596
chr1	63288208	63400830
chr1	65329180	66721347
chr1	67141298	69348189
chr1	69741433	70650823
chr1	70724208	70855701
chr1	71188906	71413345
chr1	75767642	76681700
chr1	78743485	79633798

chr1	80698356	80812030
chr1	80871186	81304745
chr1	83175061	84710421
chr1	95895750	96251134
chr1	96860270	97585510
chr1	97649529	99485029
chr1	100114467	101759014
chr1	101852774	106993251
chr1	107257942	107488078
chr1	108761989	109593180
chr1	110088631	114627348
chr1	115296247	118076358
chr1	118367486	119471376
chr1	122705036	123145030
chr1	123435862	127251194
chr1	127767831	128474785
chr1	129167657	129309020
chr1	129369160	129621426
chr1	130338551	131122652
chr1	131367385	132537622
chr1	134545432	134651655
chr1	138948338	139452055
chr1	141054627	144411636
chr1	144641591	145444908
chr1	145642012	146142591
chr1	146207363	147682856
chr1	148359083	151657452
chr1	152114799	152868188
chr1	155421403	155539628
chr1	156023837	156461554
chr1	156571160	156839417
chr1	159836200	161150172
chr1	164702094	165025861
chr1	165095642	165291636
chr1	165386148	166111285
chr1	166641514	167105894
chr1	168251695	168688218
chr1	168970024	169125432
chr1	169327936	170105788
chr1	170717534	171602382
chr1	171735266	172037044
chr1	172179783	172310372
chr1	173429815	173928554
chr1	174472344	174589056
chr1	174972286	175317650
chr1	175885652	178627921

chr1	180388768	181406639
chr1	183217628	183736446
chr1	183820243	184011457
chr1	184484487	184825716
chr1	186754249	186977331
chr1	187245773	188031019
chr1	188574264	189038120
chr1	189144364	191494002
chr1	191983318	192637583
chr1	193988400	194233624
chr1	194318074	194519024
chr1	195388491	196405560
chr1	196927533	197158929
chr10	3341066	4484760
chr10	4684117	5046372
chr10	5292830	5729579
chr10	6399406	7228769
chr10	7707772	8562594
chr10	8628656	9200033
chr10	9344559	10164943
chr10	10268772	10892469
chr10	11034890	11790093
chr10	12753549	13101268
chr10	14442901	14624825
chr10	14932515	15211183
chr10	18300556	18516207
chr10	20092659	20965318
chr10	22496098	23452401
chr10	28295936	29006497
chr10	29139230	29248740
chr10	29591222	29955433
chr10	30567782	31021125
chr10	31376788	32426204
chr10	33427517	33986029
chr10	34392076	36150063
chr10	36201018	36790468
chr10	37384410	39220905
chr10	40292865	40517737
chr10	40663042	40958429
chr10	44883406	47502119
chr10	48710835	49088342
chr10	49440758	50243180
chr10	50483045	51384312
chr10	52189080	53060255
chr10	53221111	53670557
chr10	54294209	57164646

chr10	57266330	57564762
chr10	58049727	58584278
chr10	61356272	61494455
chr10	62880492	63931786
chr10	64581673	65303207
chr10	69808957	69979839
chr10	70873305	74101975
chr10	74312055	74495532
chr10	78099518	78968588
chr10	82394516	82786395
chr10	83142911	83598125
chr10	84680882	85135539
chr10	86319082	87089221
chr10	87938347	88171895
chr10	88282722	88810638
chr10	89265509	90476368
chr10	90707289	92475194
chr10	93717139	93861675
chr10	94265634	94511980
chr10	96914979	97567179
chr10	99647586	101930567
chr10	102237804	107233295
chr10	108139110	110383538
chr10	111463070	114648885
chr10	114915204	115118981
chr10	115309866	115485299
chr10	115780159	115926286
chr10	116050606	116332606
chr10	116640449	116740812
chr10	117322103	118228045
chr10	118496765	119532278
chr10	120426439	120795307
chr10	121051117	122446818
chr10	122603233	126350089
chr10	128465419	129958117
chr11	6722867	7107617
chr11	8891353	11698397
chr11	11890093	12065221
chr11	12428085	16379048
chr11	16868342	17033310
chr11	17164263	18801049
chr11	18944636	19665526
chr11	21498715	21990810
chr11	23843239	28695745
chr11	29793210	29944352
chr11	30245705	30621308

chr11	33057505	33479259
chr11	33821001	35450124
chr11	35845035	38516073
chr11	38729239	39987444
chr11	40631910	43169313
chr11	44377998	45565694
chr11	45756055	48567559
chr11	49119102	49357300
chr11	50511476	51037190
chr11	51118141	51331207
chr11	52442473	53042859
chr11	55343029	57266986
chr11	58170360	58680125
chr11	62491768	63626905
chr11	63753000	65018088
chr11	65640119	66806103
chr11	66892213	67224964
chr11	67500082	67617708
chr11	70879134	71801584
chr11	73149279	74195763
chr11	80662892	81188708
chr11	81267357	81755256
chr11	82037530	82568558
chr11	88965771	90440315
chr11	90558515	93698169
chr11	96257780	96589279
chr11	99632383	99861556
chr11	103324286	103721397
chr11	103884097	104082099
chr11	104537978	104804689
chr11	105168945	105760147
chr11	107078445	107275363
chr11	107529068	107863389
chr11	108283617	109174580
chr11	110906129	112658187
chr11	113177187	113328500
chr11	113646228	114476846
chr11	114908370	115014046
chr11	118567596	118787176
chr12	3960580	4083136
chr12	5017104	7794515
chr12	9061781	9539309
chr12	10240672	10861413
chr12	11013672	11266384
chr12	11347982	12813318
chr12	13312798	13623590

chr12	14593061	15917507
chr12	16136615	16486615
chr12	25474815	25582318
chr12	26585257	27108959
chr12	27228274	27705396
chr12	28986787	29233988
chr12	30006640	31540742
chr12	34122388	34690928
chr12	35160509	35325514
chr12	36625731	36834348
chr12	36911274	40686818
chr12	42053696	42293634
chr12	42675687	42960472
chr12	43104780	45318817
chr12	45405494	45908380
chr12	46290750	50427235
chr12	51354179	52399961
chr12	54054354	55197380
chr12	56647082	56794603
chr12	57498509	58555327
chr12	58661488	59901599
chr12	60350189	61245209
chr12	61432536	64360285
chr12	64417497	66002705
chr12	66225934	66533252
chr12	66648205	70228089
chr12	71266037	71498122
chr12	72283920	72874051
chr12	73179215	73553741
chr12	73629428	73795172
chr12	73919944	74095711
chr12	74625016	74810419
chr12	75104635	76495860
chr12	82109494	82553068
chr12	82660340	83077522
chr12	83643491	84246725
chr12	84706896	84839343
chr12	87640247	88091386
chr12	89552584	92212503
chr12	92322364	92779066
chr12	93153107	96502930
chr12	96906862	99883004
chr12	100661301	101125626
chr12	101180344	101346661
chr12	102352312	103104891
chr12	104107341	105063971

chr12	105304931	105437253
chr12	105514540	105916981
chr12	106037658	106143971
chr12	106323310	106749878
chr12	107110359	109301999
chr12	110216947	111047582
chr12	113244299	113467716
chr12	114465820	115910043
chr12	116043128	116304669
chr12	117047345	117388576
chr12	117851748	119883407
chr12	120061601	120583311
chr12	120644196	121257242
chr13	4407450	4577183
chr13	6696075	8802938
chr13	9867659	10966416
chr13	11175552	12493843
chr13	14775019	15638720
chr13	18454655	18744221
chr13	18987234	19288429
chr13	20388176	21232054
chr13	21634941	21762981
chr13	22204047	22773356
chr13	22918841	23328719
chr13	25287475	25689726
chr13	36464311	37512488
chr13	38352355	38618775
chr13	38779128	40601276
chr13	41151186	41312463
chr13	43675651	44775872
chr13	45136696	45632843
chr13	46318466	46764248
chr13	47250781	48555714
chr13	50488724	50611081
chr13	51468241	51712405
chr13	53182198	53405790
chr13	54251711	54505894
chr13	56336722	56459662
chr13	56917942	58059056
chr13	58778815	59481898
chr13	60241451	60445183
chr13	61099055	61730785
chr13	64529772	65409381
chr13	67076779	67196173
chr13	67875397	68013637
chr13	68697755	69540626

chr13	69793263	70703122
chr13	71173369	73463423
chr13	74080598	74400514
chr13	76292890	78792122
chr13	78883805	81005479
chr13	81176866	81786119
chr13	81897267	85330309
chr13	85677253	89579244
chr13	89685416	89794165
chr13	93493878	93941869
chr13	93996146	94146024
chr13	94231542	94541135
chr13	95638352	96061820
chr13	96149687	97119998
chr13	101635500	102263156
chr13	103425662	103638773
chr13	103713673	104501170
chr13	105576009	105976914
chr13	106179920	107687031
chr13	107845478	108260267
chr13	109561557	109964352
chr13	110658024	111155222
chr13	112636826	113037822
chr13	113310654	114336217
chr13	114434791	115049477
chr13	116052890	117684837
chr13	117964583	118417315
chr13	118568375	120169204
chr14	9301037	12287279
chr14	13017589	13818978
chr14	13889733	14787718
chr14	14982730	15269748
chr14	15668928	17032544
chr14	17535883	18034800
chr14	20445376	20859257
chr14	22941487	24545299
chr14	28411297	30726600
chr14	30982559	31275874
chr14	33529515	33744771
chr14	33816118	34009486
chr14	34356997	34830860
chr14	35620504	37351237
chr14	37996198	41697444
chr14	45361210	45878349
chr14	49217099	49510068
chr14	49862656	51398492

chr14	51733037	52169393
chr14	52943977	53385238
chr14	54156327	54640905
chr14	55828540	56028567
chr14	56952927	57135411
chr14	58575408	58783778
chr14	59153509	59767512
chr14	60223128	60837747
chr14	60935849	61162113
chr14	63011543	63145424
chr14	67858009	68166674
chr14	68658017	69823020
chr14	71316847	72065277
chr14	72424204	72945675
chr14	72999589	73325096
chr14	75462818	75637356
chr14	75797143	76077024
chr14	77466461	78733591
chr14	78950155	79149313
chr14	80057903	80332756
chr14	80507115	80862096
chr14	81142925	83279932
chr14	83483354	84836893
chr14	84900686	87484161
chr14	87829210	91702819
chr14	92511967	93182313
chr14	93689688	94087311
chr14	94137979	96956251
chr14	97265490	99431891
chr14	100298658	101763517
chr14	101816688	101993522
chr14	102556999	103432136
chr14	103919130	105510724
chr14	106320961	108271555
chr14	108709094	117277689
chr14	117447950	118493144
chr14	118577165	118875424
chr14	119428062	120607159
chr14	120884290	121156845
chr14	123735928	125178134
chr15	4007244	5039237
chr15	5738477	6135762
chr15	7169355	7382392
chr15	8437294	8841221
chr15	9056433	10530800
chr15	10682074	11621197

chr15	11691796	11875779
chr15	12307911	12685780
chr15	13888765	16181601
chr15	16680779	20561707
chr15	21152468	24538318
chr15	24599101	25001391
chr15	26020188	27258599
chr15	28101914	31068309
chr15	31557767	32877273
chr15	33061479	33224276
chr15	34498753	34906562
chr15	35867069	36059896
chr15	38796060	39591600
chr15	39659478	39986886
chr15	40426525	40990864
chr15	41719358	50047569
chr15	50103391	50953037
chr15	51004254	51508748
chr15	51842192	52294551
chr15	52347925	52587951
chr15	53567976	54273384
chr15	54511528	54865934
chr15	55142047	55908573
chr15	55983520	57348091
chr15	64515081	64783121
chr15	65625870	66314340
chr15	67308044	67971918
chr15	68233390	69105966
chr15	69301431	72667478
chr15	74599685	74726639
chr15	82398052	82610465
chr15	83452867	83987684
chr15	84038715	84273836
chr15	86279684	87398734
chr15	87873268	88465481
chr15	89338495	90055459
chr15	90290606	90400657
chr15	90474080	93092686
chr15	93281962	94370426
chr15	94437231	95572026
chr15	96342520	97477919
chr15	98016822	98332273
chr15	100498383	100893154
chr15	101376490	101767113
chr15	102652035	102923684
chr15	103257417	103465445

chr16	5502770	6835665
chr16	7014352	7541510
chr16	7744375	8596161
chr16	9343637	10388201
chr16	12185957	12999829
chr16	16671775	16810965
chr16	19002262	20050264
chr16	21116723	21308338
chr16	21442358	21719816
chr16	22404258	22524769
chr16	25650693	26181532
chr16	26418925	26574071
chr16	26633874	27335510
chr16	27484329	29578554
chr16	35171655	35273727
chr16	35409674	35683637
chr16	36126741	36830814
chr16	36949637	37487974
chr16	39187070	42444791
chr16	42606404	42819913
chr16	44236568	44675081
chr16	45485493	45589723
chr16	46123660	46509712
chr16	46983012	49504773
chr16	50528034	50658889
chr16	50786584	51965658
chr16	52503560	55185248
chr16	56159606	57104903
chr16	57740741	58266150
chr16	58698163	64759610
chr16	64881079	69698730
chr16	69805989	71285856
chr16	72218539	72607632
chr16	72959325	75727853
chr16	75798141	76134773
chr16	76579897	76959460
chr16	77703524	78181101
chr16	78420598	84598611
chr16	85307955	85630047
chr16	87528759	87640394
chr16	87765210	88362011
chr16	88563078	90204832
chr16	90303192	90916915
chr16	91075181	91322783
chr16	93316374	93535000
chr16	94934788	95818965

chr16	96392824	96585569
chr16	96645412	97474141
chr17	3561382	4671374
chr17	5445792	5765549
chr17	7645901	7940377
chr17	8611666	9956528
chr17	10550534	10873814
chr17	10963028	12241932
chr17	12752849	12858997
chr17	14345236	14977648
chr17	15974655	17112270
chr17	17547265	17653376
chr17	18034585	21114645
chr17	21361796	21536908
chr17	21844008	22049218
chr17	22474686	23104516
chr17	23292477	23627844
chr17	30840183	31170917
chr17	32737460	32838122
chr17	36459978	36924489
chr17	37220328	39481104
chr17	40337106	42889944
chr17	43585907	43763697
chr17	43878923	44177473
chr17	48258309	48456635
chr17	48558407	49557129
chr17	49641348	50501236
chr17	50696322	51127379
chr17	51345007	53615839
chr17	54285103	56044176
chr17	57514430	59197991
chr17	59407949	62114856
chr17	63233040	63623853
chr17	65000054	65897521
chr17	66966779	71022753
chr17	72042446	73128475
chr17	76425082	78583089
chr17	78869494	79015758
chr17	79476665	79943164
chr17	81433805	82252823
chr17	82737697	83554403
chr17	83943831	84058559
chr17	84875088	85102878
chr17	85502333	86253973
chr17	86335517	86486801
chr17	86990264	87124056

chr17	88479947	88832932
chr17	89054008	91348901
chr17	91419015	91737083
chr17	91884470	95254916
chr18	3181133	3334145
chr18	7038079	7552535
chr18	9576038	9909111
chr18	12636939	12738208
chr18	13165403	13525645
chr18	13707642	14646591
chr18	15013142	16394176
chr18	17328337	19758141
chr18	20080760	20478260
chr18	21485613	23900271
chr18	26458693	31683571
chr18	32649572	33337387
chr18	34028875	34144085
chr18	37037161	37918393
chr18	40399960	42370631
chr18	43605369	44004398
chr18	44065315	44534992
chr18	45011307	46110218
chr18	46981665	49893273
chr18	50310387	51274712
chr18	51525467	53292667
chr18	53599615	53801815
chr18	54157747	54738196
chr18	55533200	56483335
chr18	58116226	59311485
chr18	59499544	60611917
chr18	62231037	62640112
chr18	62717505	63791092
chr18	66719782	67354328
chr18	68028091	69503415
chr18	69554235	69851799
chr18	70819288	71725794
chr18	71829460	73705596
chr18	74451227	74585118
chr18	74726124	74889177
chr18	75929523	76337367
chr18	76772324	77160220
chr18	77220595	77837544
chr18	78567743	79366522
chr18	79450605	80263195
chr18	81179770	82614899
chr18	83183354	84247770

chr18	84315929	84454628
chr18	85115458	85534525
chr18	85623212	87943695
chr18	88240254	88530372
chr18	89233685	89576738
chr18	89642171	90390091
chr19	7683384	7929213
chr19	8447990	8726497
chr19	11231255	11396474
chr19	12113822	12494818
chr19	12917983	13531486
chr19	13588084	14531543
chr19	14786777	15396996
chr19	15461353	15955278
chr19	17487983	18628326
chr19	20839635	21132034
chr19	21403228	21622100
chr19	21993355	22985868
chr19	23392083	23564178
chr19	23758517	23918161
chr19	25018857	26807914
chr19	30299411	32141663
chr19	33099069	33724977
chr19	33918952	34224080
chr19	35035368	36159134
chr19	36348932	36705845
chr19	38523365	38857608
chr19	39048726	39657410
chr19	40000801	40384434
chr19	40679030	41262489
chr19	42396637	42554078
chr19	42862779	43492805
chr19	48007171	53093972
chr19	53198502	53338092
chr19	54169427	54634355
chr19	57738464	58474803
chr19	58565442	59358618
chr19	59456278	59821810
chr19	60272060	60528469
chr2	4600004	4746515
chr2	7160043	8700950
chr2	9244409	9539283
chr2	10065678	11244159
chr2	11508872	11619011
chr2	11762607	12341927
chr2	12841110	13163977

chr2	14243591	14445802
chr2	14659773	14956339
chr2	15138276	16256900
chr2	16880662	17917140
chr2	18653890	18799940
chr2	19018321	19437668
chr2	19653729	20344556
chr2	21228284	22471284
chr2	22608079	22742578
chr2	23060489	24099689
chr2	35642945	35783070
chr2	36173208	37180794
chr2	39111338	44447815
chr2	44539956	44672784
chr2	45018955	45320205
chr2	45536802	48154894
chr2	49973428	50078973
chr2	51310053	51698860
chr2	51962543	52231363
chr2	52358925	52516698
chr2	53084717	57012815
chr2	57211427	58298726
chr2	58610245	58819892
chr2	61635283	62478275
chr2	63234923	64893950
chr2	65159611	65892023
chr2	66049040	66379056
chr2	66548318	68635137
chr2	69021761	69129619
chr2	69322563	69457777
chr2	69905492	70457759
chr2	71788455	71895810
chr2	73771634	74981610
chr2	75869090	77059700
chr2	77179079	77874899
chr2	78779362	79345063
chr2	79515425	80074135
chr2	80204817	80324705
chr2	80491926	81971646
chr2	82186598	83441117
chr2	83714375	84244978
chr2	85100886	87690580
chr2	88134124	90138924
chr2	94303918	95299592
chr2	96072391	100281253
chr2	100623441	101485262

chr2	104037462	104144218
chr2	105061823	106798932
chr2	107207459	107814096
chr2	110048404	112045630
chr2	112340785	113148484
chr2	113522043	113628463
chr2	113793025	115295760
chr2	115880080	116731443
chr2	121452712	121755959
chr2	122228197	122479149
chr2	122629281	124445034
chr2	125108582	125380687
chr2	127744287	127894669
chr2	129148821	130094431
chr2	132174966	132316094
chr2	132821980	133267277
chr2	133731289	136432606
chr2	136521906	136666460
chr2	138094580	139740508
chr2	140021846	140498046
chr2	140777227	142439613
chr2	142907503	143674428
chr2	144419479	145045786
chr2	145106317	145412903
chr2	146476172	146768986
chr2	146913713	147265827
chr2	148756020	150571199
chr2	150783015	150999942
chr2	151432831	151744901
chr2	151865839	152001997
chr2	152240767	152418718
chr2	153724800	154154568
chr2	158462940	158575954
chr2	158681511	160436083
chr2	162394862	162747976
chr2	162923315	163184661
chr2	163685421	163791335
chr2	163995450	164184876
chr2	166066104	166554901
chr2	168432788	169912124
chr2	170084473	170209058
chr2	170354069	172150816
chr2	172326495	172812602
chr2	172898934	173022755
chr2	173167891	173433117
chr2	173631883	174229031

chr2	174323151	174775580
chr2	177829754	179621693
chr3	3267739	3717667
chr3	4227567	4630424
chr3	5605171	7318353
chr3	7782376	8629250
chr3	10478693	12707165
chr3	12796893	13831867
chr3	16167070	19007344
chr3	19466762	19586447
chr3	20001057	21179416
chr3	22129403	22975106
chr3	23080886	23460602
chr3	23530931	24169317
chr3	24266944	26931997
chr3	28855628	29644306
chr3	30125482	30383466
chr3	31049550	31502026
chr3	31624566	32124679
chr3	32669621	33826984
chr3	34018563	35601158
chr3	36824427	37107565
chr3	38743866	40369541
chr3	41446006	42383989
chr3	42802216	50195262
chr3	52802656	53195567
chr3	53276130	54256852
chr3	54599267	56749418
chr3	58566540	59255878
chr3	59340534	59880745
chr3	60477308	61028478
chr3	61276105	61902400
chr3	62322047	62986255
chr3	65972759	67856204
chr3	69335167	69460723
chr3	69747267	70009202
chr3	70171966	75185539
chr3	75390086	75516043
chr3	75811641	78744989
chr3	79461031	79595364
chr3	79869077	80772122
chr3	81699057	83592772
chr3	85043874	85284526
chr3	86633255	86780315
chr3	87113808	87265629
chr3	91744835	91908021

chr3	92533416	92840450
chr3	92899170	93284265
chr3	98511297	98944746
chr3	99045695	99961513
chr3	100121500	100398726
chr3	105041338	105471664
chr3	105757227	106237540
chr3	106645148	107040686
chr3	109568503	113029298
chr3	113236149	115582416
chr3	116850894	119386610
chr3	119870111	120964123
chr3	122402195	122558555
chr3	123108667	123213631
chr3	123425428	124987177
chr3	125085194	125557153
chr3	126681983	127444801
chr3	127685739	128219698
chr3	128308122	129230430
chr3	129286273	129702355
chr3	129797035	130382259
chr3	130695501	130914187
chr3	131452389	131671064
chr3	133281620	133698575
chr3	133885603	134657462
chr3	135421319	136091520
chr3	136511309	137156533
chr3	137845272	138076429
chr3	138420864	138662019
chr3	138718962	139905601
chr3	141172548	141878675
chr3	142161865	142312456
chr3	142607627	143537601
chr3	145605239	145985708
chr3	146588029	150948003
chr3	151024826	151746548
chr3	152442018	157571468
chr3	157722825	159467776
chr4	3891008	4504293
chr4	4750151	6211741
chr4	8733812	9537428
chr4	9641777	10800791
chr4	11257199	11374153
chr4	12153110	14749124
chr4	14858288	15864012
chr4	16157385	19453903

chr4	19680682	20719618
chr4	20811568	21648929
chr4	21836361	22270825
chr4	22349171	24431782
chr4	24486726	26710195
chr4	29150749	30344277
chr4	32884825	33066041
chr4	33965270	34213194
chr4	35194731	40052692
chr4	40251844	40595921
chr4	44431057	44583691
chr4	46763449	46980672
chr4	47537519	48033981
chr4	48566317	49146182
chr4	49623998	53136284
chr4	56996350	57098511
chr4	57365229	57633713
chr4	58016051	58780499
chr4	64068534	64402878
chr4	64504334	66393654
chr4	66671382	70007683
chr4	70249745	71733804
chr4	71901350	73905138
chr4	73959558	76873586
chr4	76974124	77852802
chr4	78078784	80507863
chr4	80795936	81127271
chr4	82480719	82812701
chr4	83212849	86191868
chr4	86535615	87147173
chr4	89227971	91638526
chr4	92104215	93367839
chr4	94325126	94595319
chr4	95615459	97379346
chr4	98410048	98513171
chr4	98889673	99490935
chr4	100703181	100807369
chr4	101043820	102166374
chr4	102252173	102760668
chr4	103029472	104640319
chr4	109626432	113001582
chr4	113149938	113875864
chr4	114013396	114578527
chr4	118413914	118641861
chr4	122229765	122457535
chr4	124962834	125642119

chr4	127083890	128106543
chr4	128246005	128355575
chr4	131064450	131359784
chr4	136227514	136389349
chr4	141719255	142690588
chr4	142801929	143094651
chr4	143733863	144416248
chr4	147575535	147753253
chr4	150430826	151154813
chr4	151738067	153297579
chr4	153386671	154212674
chr5	4208786	5150677
chr5	5780515	8621001
chr5	9168653	10655187
chr5	11940347	12572247
chr5	12783955	13608799
chr5	14285370	14896795
chr5	15807274	16408852
chr5	17789822	20294825
chr5	20817842	20952981
chr5	21315345	21997670
chr5	22101041	22801589
chr5	25107118	25409203
chr5	26469833	28013788
chr5	28117065	28246409
chr5	28460823	28587071
chr5	28902008	29379858
chr5	37777265	38112350
chr5	38646521	38842949
chr5	39000890	40007737
chr5	40386769	42175961
chr5	42555426	43408246
chr5	43662009	44052127
chr5	44400393	44570427
chr5	44694793	45250935
chr5	45461397	45810386
chr5	45936942	46186919
chr5	46269406	47835826
chr5	48008738	50390279
chr5	50451248	51499233
chr5	51656338	51882416
chr5	52150139	52491838
chr5	52611874	52888385
chr5	54552010	63048579
chr5	63200371	64165831
chr5	67108538	67613436

chr5	67774370	68668452
chr5	68868332	72562533
chr5	73864712	74357167
chr5	74605521	74891882
chr5	75019927	75191227
chr5	75499283	76470896
chr5	76981899	77286666
chr5	77459502	77624518
chr5	78208598	81052160
chr5	82536326	85501663
chr5	85608782	86434671
chr5	86638795	87188888
chr5	87621380	87946082
chr5	88051187	88972224
chr5	96745507	97028921
chr5	98646531	99327487
chr5	99392859	99506169
chr5	100008444	100364693
chr5	100473115	100727076
chr5	101700897	102237967
chr5	102587676	102898505
chr5	103273784	104078395
chr5	104507989	104774629
chr5	105346044	105491786
chr5	106356086	106799779
chr5	107164109	107389976
chr5	109390459	110114338
chr5	111870418	112296947
chr5	112844539	113484928
chr5	113651649	114170743
chr5	116518703	116823453
chr5	116936510	117455077
chr5	117870643	118228238
chr5	119321759	120491191
chr5	126025841	126998763
chr5	127060165	129024104
chr5	129129304	129487473
chr5	129551972	129911103
chr5	130793088	131651813
chr5	131726224	134547901
chr5	137177563	137284900
chr5	138659548	139014871
chr5	141322089	142823331
chr5	145178629	145440713
chr5	147134030	147466180
chr5	148704083	149150384

chr5	149529308	149717884
chr5	149858458	151382902
chr5	152148994	152534864
chr6	3490119	5171263
chr6	5575266	7763377
chr6	7876052	11762597
chr6	12035579	12246994
chr6	14181914	15626831
chr6	15815584	16941730
chr6	18773514	21766392
chr6	21913158	22275420
chr6	22585047	24071403
chr6	24193813	24541064
chr6	24657939	28360858
chr6	31914583	32156490
chr6	32340695	32650197
chr6	35647615	37257393
chr6	39733250	40317079
chr6	40425651	40914746
chr6	41016872	41431451
chr6	41543618	42166448
chr6	42544271	43178438
chr6	43679215	47074963
chr6	51145534	51276930
chr6	55393256	56625846
chr6	56891203	57397098
chr6	57513251	57617354
chr6	57822000	58475859
chr6	59100383	59763622
chr6	59893632	60658323
chr6	60831717	64983557
chr6	65083304	66791919
chr6	67257070	67557196
chr6	67711346	68919320
chr6	69641595	70267526
chr6	72996495	73100732
chr6	73265605	78598235
chr6	78858390	81745821
chr6	81955146	82598295
chr6	92267675	92576436
chr6	95147101	95530196
chr6	95703036	97064794
chr6	97493975	97696746
chr6	97877799	98517592
chr6	99508791	99751487
chr6	100852304	106610534

chr6	107007914	107991308
chr6	108815762	112125883
chr6	112354027	112581470
chr6	113735944	114231899
chr6	116372777	117745500
chr6	118430008	118876185
chr6	119008416	119247206
chr6	121223246	122088446
chr6	122195422	122711002
chr6	122836603	123292940
chr6	123612496	124348389
chr6	125581550	125938001
chr6	126007238	126999855
chr6	127110279	127871711
chr6	128535946	128712921
chr6	131384067	132050725
chr6	132283967	133125903
chr6	133490603	133859549
chr6	135391952	136436796
chr6	136511782	139524195
chr6	139794962	140313385
chr6	140638856	142080946
chr6	142332276	143319809
chr6	143633500	144580812
chr6	144713512	145154787
chr6	145317325	145607331
chr6	147057505	147651838
chr6	147736671	148721882
chr6	149362182	149516399
chr7	4167641	4370694
chr7	5097797	5342396
chr7	6155789	7154154
chr7	10709960	11351052
chr7	12223065	13436313
chr7	13658434	14273834
chr7	14656892	15240900
chr7	18221416	18807986
chr7	24083537	24428083
chr7	24553630	25033190
chr7	26722995	27604824
chr7	31564031	31704181
chr7	34389305	34858569
chr7	36642644	38689924
chr7	46852234	47915854
chr7	48015304	48377413
chr7	48571229	49865883

chr7	54437157	55033841
chr7	55141873	55977659
chr7	57146298	59088019
chr7	59216201	61094410
chr7	61183958	63046527
chr7	63476072	65608469
chr7	66308108	66481411
chr7	67067106	67734118
chr7	67958623	68569576
chr7	69366738	70894407
chr7	71549106	72399205
chr7	73983827	74202539
chr7	75420568	77136901
chr7	77504151	79637648
chr7	80300966	80519500
chr7	82311863	82456465
chr7	83088110	84141772
chr7	84301633	85896713
chr7	88768374	88896457
chr7	88975197	89164903
chr7	89248732	89807063
chr7	89929553	90949100
chr7	91538459	91715482
chr7	91886549	94298831
chr7	94467061	95353364
chr7	95688212	96203475
chr7	97843971	98785235
chr7	100096256	100504961
chr7	101226359	102346272
chr7	102464946	104064738
chr7	109684953	109971164
chr7	110077093	111371330
chr7	111430424	112586330
chr7	113385088	115143705
chr7	115485384	116083138
chr7	116137092	116621717
chr7	118443338	119102958
chr7	120941270	121133024
chr7	121446275	122707534
chr7	122821974	123222821
chr7	123718505	124863492
chr7	126184558	126977907
chr7	127084190	127266997
chr7	127344169	127771768
chr7	128238181	129077531
chr7	129320984	130049234

chr7	130604458	132589114
chr7	135939223	136771317
chr7	138197877	138489016
chr7	138587322	139440496
chr7	139643863	139759506
chr7	142405461	142656365
chr7	142944971	144426641
chr7	144660621	146020669
chr7	146123637	146387685
chr7	146782598	147137007
chr7	147339999	148012300
chr7	149662118	150179333
chr7	150313127	150427995
chr7	150518130	150649713
chr8	3697743	4177557
chr8	5341859	6003220
chr8	8735509	8990532
chr8	9213134	9945217
chr8	14120902	14232037
chr8	15123215	17500644
chr8	18908630	19654603
chr8	22924437	23075722
chr8	23362572	23481158
chr8	24440113	24645440
chr8	24708004	25459259
chr8	25729469	25945613
chr8	28439059	32156198
chr8	33916234	34170012
chr8	36589567	36914434
chr8	37135830	40033725
chr8	40485839	41370684
chr8	41868968	42087564
chr8	42559088	43076839
chr8	43276976	45712791
chr8	48061601	48568957
chr8	49120245	55465234
chr8	55747766	56192094
chr8	58084772	58533540
chr8	59063974	59904967
chr8	60028524	63340116
chr8	64180680	66119095
chr8	67518373	70201813
chr8	70890098	71235001
chr8	75969255	77515078
chr8	77855265	79407012
chr8	79669689	80022984

chr8	80126886	81024739
chr8	82294139	83476686
chr8	83570085	84215921
chr8	85503754	86067834
chr8	89629572	90618570
chr8	90938453	91170647
chr8	92346781	93282646
chr8	93692861	93817253
chr8	94944893	96374109
chr8	98046427	98159235
chr8	98477364	106714652
chr8	110143004	112082195
chr8	112567897	113129238
chr8	114585400	116672220
chr8	116811172	117068462
chr8	117278741	117995529
chr8	118644212	119427337
chr8	120006224	120171411
chr8	120311987	120766014
chr8	120848947	121884918
chr8	123211037	123451927
chr8	123603984	123981056
chr8	126889054	126990767
chr8	127690823	128937107
chr8	131281465	131708489
chr9	3326524	5955102
chr9	6757730	6951801
chr9	7301142	7520782
chr9	8063003	9019779
chr9	9380674	13136146
chr9	14610902	14836902
chr9	15161004	18388772
chr9	18568813	19498086
chr9	19627588	20219338
chr9	22529752	23346201
chr9	23804910	24895884
chr9	25425641	26773371
chr9	26859406	28733577
chr9	28874640	29008026
chr9	29248158	30141065
chr9	31770789	32407152
chr9	32659578	33282045
chr9	34306208	34663932
chr9	35383562	36496961
chr9	36594019	36978558
chr9	37739668	40016945

chr9	40169501	40553414
chr9	40650324	40804018
chr9	42387563	42679264
chr9	47753514	48263500
chr9	48863121	49219927
chr9	51175488	51706032
chr9	52137324	52979058
chr9	53526403	54295522
chr9	67168296	67305516
chr9	67564116	69131267
chr9	69412877	69688286
chr9	75451572	75783205
chr9	75938845	76268601
chr9	76447529	77267228
chr9	78498633	78789113
chr9	79676314	79873893
chr9	81846482	82733495
chr9	82884356	83387505
chr9	83520296	84140074
chr9	84427046	84884553
chr9	85224558	85598804
chr9	85674548	86329362
chr9	86692428	88141586
chr9	89111009	89706746
chr9	90212774	92089703
chr9	92376334	93870467
chr9	94412974	95351005
chr9	97096694	98041471
chr9	98482741	98891121
chr9	99664285	100306414
chr9	101192989	102486860
chr9	103303525	103946973
chr9	104175681	106042743
chr9	106409231	106533017
chr9	106805472	107148385
chr9	109279734	109728130
chr9	111198431	111583389
chr9	112016645	113544615
chr9	113901009	114034118
chr9	114301358	114416076
chr9	114901103	115085915
chr9	115270864	116001090
chr9	116295095	117816121
chr9	117870627	118383466
chr9	118484899	118809344
chr9	119373254	119785998

chr9	120032632	120483828
chr9	121383906	121546329
chr9	122336953	122727675
chr9	123834220	124061522
chrX	5185664	5921069
chrX	6121117	6703197
chrX	8417284	8735382
chrX	9633858	9763960
chrX	13488098	16060192
chrX	19598736	19892850
chrX	20585396	22912488
chrX	23436067	23665858
chrX	33248984	33490815
chrX	36043751	39141252
chrX	39891493	40717183
chrX	40851410	45256345
chrX	46038516	46278817
chrX	46754748	46925992
chrX	47101946	47310806
chrX	47421985	47890500
chrX	48018986	49166051
chrX	49268346	49501963
chrX	49657300	50228556
chrX	50970451	51301941
chrX	53404652	53819012
chrX	54058935	54616193
chrX	54691175	54860322
chrX	54910458	55152159
chrX	55328660	56289182
chrX	56394870	57586612
chrX	57671009	59869128
chrX	59980223	62973187
chrX	63495661	63926345
chrX	64583628	64987428
chrX	66550097	67388056
chrX	72428364	72578856
chrX	73384581	74293642
chrX	75047612	75733610
chrX	75840853	82277500
chrX	83172263	83828990
chrX	83921947	88648428
chrX	89224024	90290826
chrX	90574720	90679168
chrX	91657347	92071765
chrX	92183582	93131194
chrX	93392920	94481823

chrX	94624998	95787033
chrX	98012708	98212737
chrX	101235458	101397140
chrX	102330377	102960936
chrX	103452338	103744173
chrX	103903983	105761228
chrX	106115342	106232613
chrX	106410576	109403380
chrX	109463762	110132019
chrX	110354248	115582135
chrX	115695237	116310084
chrX	116364862	120107134
chrX	122949551	125881670
chrX	127221249	130607590
chrX	130898674	131022713
chrX	131462231	131611382
chrX	132100678	132719912
chrX	132842630	133431596
chrX	133684396	135093842
chrX	136634216	136758394
chrX	139509844	139879861
chrX	140220914	141848981
chrX	141991231	143665758
chrX	143828189	144167250
chrX	149338676	149610478
chrX	150144830	150417282
chrX	150604054	151358573
chrX	151806425	153932120
chrX	154064586	155726016
chrX	155823306	155932109
chrX	156394417	156516357
chrX	157486034	159148373
chrX	159385591	160354442
chrX	160493033	161362720
chrX	162061338	162569265
chrX	163851376	165359890
chrY	249421	776520
